# Low Rates of Dual-Site and Concordant Oral-Cervical Human Papillomavirus Infections and Cancers: A Systematic Review

**DOI:** 10.3389/fonc.2022.848628

**Published:** 2022-03-29

**Authors:** Kelsey H. Jordan, Chloe M. Beverly Hery, Xiaochen Zhang, Electra D. Paskett

**Affiliations:** ^1^Division of Population Sciences, Comprehensive Cancer Center, The Ohio State University, Columbus, OH, United States; ^2^Division of Epidemiology, College of Public Health, The Ohio State University, Columbus, OH, United States; ^3^Arthur G. James Cancer Hospital and Richard J. Solove Research Institute, Columbus, OH, United States; ^4^Division of Cancer Prevention and Control, Department of Internal Medicine, College of Medicine, The Ohio State University, Columbus, OH, United States

**Keywords:** female, (human) papillomavirus (HPV) infection, oropharynx, cervix (uteri), cancer, epidemiology, systematic review

## Abstract

**Objective:**

The oral-cervical human papillomavirus (HPV) infection/cancer relationship is not well established. Oral-cervical HPV studies were reviewed to assess dual-site occurrence, HPV type concordance, and study quality/deficiencies.

**Methods:**

PubMed, EMBASE, Ovid Medline, and Web of Science were searched between 1/1/1990 and 8/10/2021 for studies investigating HPV infections/cancers and type concordance between the oral cavity/oropharynx and cervix. Dual-site and concordant HPV infection rates were summarized as percentages; cancer diagnoses studies were summarized using standardized incidence ratios (SIR). The Quality Assessment Tool for Quantitative Studies (QATQS) evaluated study methodology.

**Results:**

One hundred fourteen papers were identified. Most were cross-sectional (n=79, 69%), involved synchronous dual-site HPV testing (n=80, 70%), did not report HPV type concordance (n=62, 54%), and achieved moderate methodological QATQS ratings (n=81, 71%). The overall dual-site infection rate averaged 16%; the HPV type concordance rate averaged 41%, among those dually-infected women. Most HPV-related cancer diagnoses studies reported increased secondary cancer risk, with SIRs generally ranging from 1.4 to 29.4 for secondary cervical cancer after primary oral cancer and from 1.4 to 6.3 for secondary oral cancer after primary cervical cancer.

**Conclusion/Impact:**

Oral-cervical HPV infections/cancers remain understudied. Future research should use stronger methodologies and HPV concordance analyses to better understand oral-cervical HPV epidemiology.

## Introduction

Human papillomavirus (HPV) is the most prevalent sexually transmitted infection (1). The virus exists in 200+ types—some more high risk (i.e., potentially malignant) than others (1). Various HPV types can infect the cervix, vagina, vulva, penis, anus, and/or oropharyngeal region, increasing the risk for the development of warts and/or cancers (1). Globally, about 630,000 incident cancers are HPV-related with most occurring in the oropharynx and cervix (1, 2). Oral HPV infections and cancer biology remain less understood than cervical HPV (3–7). Cervical HPV infection is clearly acquired through vaginal intercourse, whereas acquisition of oral HPV, potentially during orogenital sex, remains uncertain, especially in women (3, 4, 8, 9). Therefore, women are disproportionately burdened with the disease, amassing 90% of all HPV-related cancers (1).

HPV can be attributed to more than 70% of oropharyngeal cancers in the United States (US) (8, 10). In 2020, there were 98,412 new oropharyngeal cancer cases worldwide (11). High-risk HPV types (e.g., HPV16) account for a substantial proportion of oral HPV cases (3). HPV tends to infect the back of the oral cavity from the base of the tongue through the esophagus, including the oropharynx and tonsils (3, 9). However, there is no routine screening for oral HPV infection and methods are less-refined for oral HPV cancer detection, resulting in later stage diagnoses and more aggressive cancer treatments (3).

Approximately 604,127 women were diagnosed with cervical cancer worldwide in 2020 (11). Essentially all cervical cancers are HPV-related (10). HPV types 16 and 31/18/33 are the first and second most common type groupings routinely identified in advanced cervical infections and cancers, respectively (5). Slow disease progression and effective screening methods, including Papanicolaou (Pap) tests, allow for opportunities to detect and treat cervical abnormalities to reduce the risk for cancer development (12).

Results from studies of dual-site oral-cervical HPV infections/cancers are inconsistent. Investigating HPV status in both oral and cervical sites in women can aid in determining how HPV is transmitted (e.g., orogenital interaction, autoinoculation, unrelated events) (4). For example, oral-cervical HPV type concordance (i.e., same HPV type(s) in both sites) would suggest a transfer of infection across sites. Whereas HPV type discordance would suggest the infections were separate. Clarity in the oral-cervical HPV+ association could improve prevention, screening, and/or treatment approaches for both diseases, ultimately reducing HPV-related cancer rates overall.

Current systematic reviews on the topic of oral HPV infections and cervical cancers have only studied the infections independently of one another. This prohibits a complete assessment of HPV type concordance between the anatomical sites. The one meta-analysis that investigated oral and cervical HPV infections estimated an HPV concordance rate of 27% (4). However, the study was limited in publication years, databases, search terms, and oral HPV data collection methodologies, including just 10 studies, and without any quality assessment.

To date, there is no published comprehensive systematic review incorporating a quality assessment of the literature that examines the potential for both oral and cervical HPV infections in women. This systematic review aims to fill significant gaps in the HPV literature regarding oral and cervical dual-site and concordance rates of HPV. In summary, there is no consensus on whether oral and cervical HPV-related infections and/or cancers are more likely to be related or unrelated events. This systematic review aims to critically assess studies with participants who have at least one HPV-related oral and/or cervical infection/cancer diagnosis, comparing any HPV types across the two biological sites, to determine if there is a higher probability that any HPV types at the two sites had concordance.

## Materials and Methods

### Literature Search Strategy

A review of the literature was conducted in PubMed, EMBASE, Ovid Medline, and Web of Science databases using variations to the search terms *oropharynx and cervix and human papillomavirus and infection or cancer*. Searches were restricted to peer-reviewed papers published from January 1, 1990 to August 10, 2021. For example in PubMed, the following terms were used:

**Table:** 

Oropharynx:	[(head and neck) OR (oral) OR (oropharyngeal) OR (oropharynx) or (oropharyn*) OR (soft and palate) OR (esophagus) or (esophageal) or (esophag*) OR (hypopharynx) or (hypopharyngeal) or (hypopharyn*) OR (larynx) or (laryngeal) or (laryn*) OR (nasopharyngeal) OR (nasopharynx) or (nasopharyn*) Or (tonsil) or (tonsillar) or (tonsil*) OR (throat)]
Cervix:	[(Cervix) or (cervical) or (cerv*)]
Human papillomavirus:	[(hpv) or (human and papilloma and virus) or (papillomaviridae) or (human and papillomavirus)]
Infection or cancer:	[(cancer) or (cancerous) or (cancer*) OR (carcinoma) or (carcinom*) OR (neoplasia) OR (neoplasm) OR (neoplas*) OR (tumor) or (tumor*) or (tumorous) OR (dysplasia) OR (intra and epithelial and neoplasia) or (intra and epithelial and dysplasia) OR (mucosal and lesion) OR (infection) or (infect*) OR (malignancy) OR (malignant) or (malignan*) OR (precancerous and lesion) OR (pre and cancerous and lesion) OR (squamous and cell and carcinoma)]
Applied search filters:	Publication date from 1990/01/01 to 2021/08/10; Humans; English

These search strategies were reproduced in each of the other three databases (**Supplementary Table**). Preferred Reporting Items for Systematic Review and Meta-Analysis (PRISMA) guidelines were used in this systematic review with respect to design and reporting.

### Eligibility Criteria

#### Inclusion Criteria

Studies were eligible for inclusion if they: involved human subjects; investigated both person-specific anatomical sites of oral cavity/oropharynx and cervix for HPV synchronously (i.e., evaluated simultaneously) or asynchronously (i.e., evaluated at different times); and were full-text papers of original research written in English. Partner studies with both men and women were included if any HPV data pertaining to women could be independently differentiated from any HPV data presented on men. Studies including participants with a positive oral HPV test or any HPV-related oral cancer (i.e., non-tobacco/alcohol-related oral cancers) were included. Oral sites could range from the oral cavity to the esophagus (both potentially HPV-associated), including the oropharyngeal region with the base of the tongue and the tonsils (both HPV-related), as long as the original study authors had justified the sites to be at least possibly oral HPV-related (3, 9). All cervical abnormalities/cancers were assumed to be HPV-related since 95–99% of cervical cancer cases involve HPV (13).

#### Exclusion Criteria

Studies were excluded if they were not relevant to within-person HPV evaluation of both oral and cervical infections (e.g., both sites but in different people, wrong biological site or cancer or population), involved only HPV infections in the oral cavity/oropharynx or cervix, not original research (e.g., reviews, abstracts, letters, commentaries, meetings, protocols), or were case reports or series (i.e., N <10).

### Data Collection, Categories, and Analyses

#### Data Extraction

Duplicate citations from the four databases were reviewed and removed. The remaining citations were divided equally, reviewed separately, and then summarized with data extraction by three study authors (KHJ, CBH, XZ). Any questions regarding inclusion were resolved by consensus among the three authors listed above.

#### Assessment of Risk of Bias and Quality of Studies

As described by Thomas et al., the Quality Assessment Tool for Quantitative Studies (QATQS) from the Effective Public Health Practice Project criteria was utilized to determine the quality of each included study (14). The assessment tool evaluates: 1) selection bias, 2) study design, 3) confounder adjustment, 4) blinding, 5) data collection methods, and 6) withdrawals and dropouts (14). All topics were evaluated for studies included in this systematic review, excluding blinding since all studies were observational in nature and no intervention or randomized control trial methods were considered for HPV evaluation in the oropharynx/oral cavity sites and/or cervix. Included papers were divided such that two authors (KHJ, CBH, or XZ) reviewed and scored the QATQS for each study independently. Each topic area evaluated received a rating of strong, moderate, or weak quality, dependent on topic-specific criteria. Studies attaining only moderate and/or strong quality topic ratings were classified as “strong”; studies with one weak quality topic rating were classified as “moderate” while studies with two or more weak quality topic ratings were classified as “weak” (14). The primary paper evaluator (KHJ, CBH, or XZ) compared the two-author ratings for inconsistencies. Discrepancies were discussed amongst authors and a consensus was reached.

#### Outcomes

Concurrent infections were defined as any HPV infection(s) occurring in both the oral cavity/oropharynx and cervix simultaneously due to synchronous site testing. If HPV infection was absent at either or both sites, then any infection was not considered concurrent. “Dual-site infections” were defined as any HPV infections occurring in both the oral cavity/oropharynx and cervix at different times due to asynchronous (i.e., non-simultaneous) testing of the two sites. Concordant infections were identified in women who shared at least one HPV type across oral and cervical sites at any time (synchronously or asynchronously) (**Table 1**).

**Table 1:** Definitions and summary statistics for a 1990-2021 systematic review of oral-cervical human papillomavirus (HPV) infection/cancer rates in women.

DEFINITIONS	CONCURRENT/DUAL-SITE INFECTIONS* Any HPV type(s)* in oropharynx/oral cavity and cervix, simultaneously (concurrent) or at varying times (dual-site)	CONCORDANT INFECTIONS *At least one identical HPV type(s)* in oropharynx/oral cavity and cervix, simultaneously or at varying times
**SYNCHRONOUS EVALUATIONS** HPV testing of oral and cervical anatomical sites at *same time*	Average: 15% Range: 0-95%	Average: 41% Range: 0-100%
**ASYNCHRONOUS EVALUATIONS** HPV testing of oral and cervical anatomical sites at *varying times*	Average 26% Range: 2-100%	Average: 39% Range 2-100%
**OVERALL ESTIMATED AVERAGE**	**16%**	**41%**

Studies investigating oral-cervical cancer diagnoses from registry data were also considered. We included studies that examined the occurrence of cervical cancer after a primary diagnosis of HPV-related oral cancer and occurrence of HPV-related oral cancer after a primary diagnosis of cervical cancer. Infections were also included here, if documented accordingly in the registries/databases.

#### Categories

Eligible studies were divided into three categories based on the timing of HPV evaluation at both sites (i.e., synchronously, asynchronously, or cancer diagnoses). Synchronous HPV evaluation studies actively collected oral and cervical samples and tested them both for HPV DNA at the same visit (with one study testing oral samples within three weeks of cervical samples). Asynchronous HPV evaluation studies either HPV-tested the oral and cervical sites at separate visits or one anatomical site was previously diagnosed with a HPV-related cancer and the other anatomical site was actively tested for HPV infection during the study. Cancer diagnoses only studies relied on data from cancer registries or medical records to determine prior primary and secondary cancer diagnoses of the oral cavity/oropharyngeal region and cervix.

#### Statistical Analyses

When individual synchronous and asynchronous studies presented sufficient results, we summarized concurrent/dual-site infection data as percentages of women with any oral-cervical HPV infections at any time. HPV type concordance data was summarized as percentages of women with the same oral-cervical HPV type(s) at any time. Overall concurrent/dual-site and concordant oral-cervical HPV infection rates were determined by averaging respective individual study percentages (**Table 1**). For cancer diagnoses studies, we summarized the overall rates of secondary cervical and/or oral cancers (number of cases per 10,000 women) and reported the standardized incidence ratios (SIR) to indicate whether the age-adjusted observed cancer cases were higher than expected for individual study populations. Results were not pooled across studies but stated as ranges.

## Results

**Figure 1:**
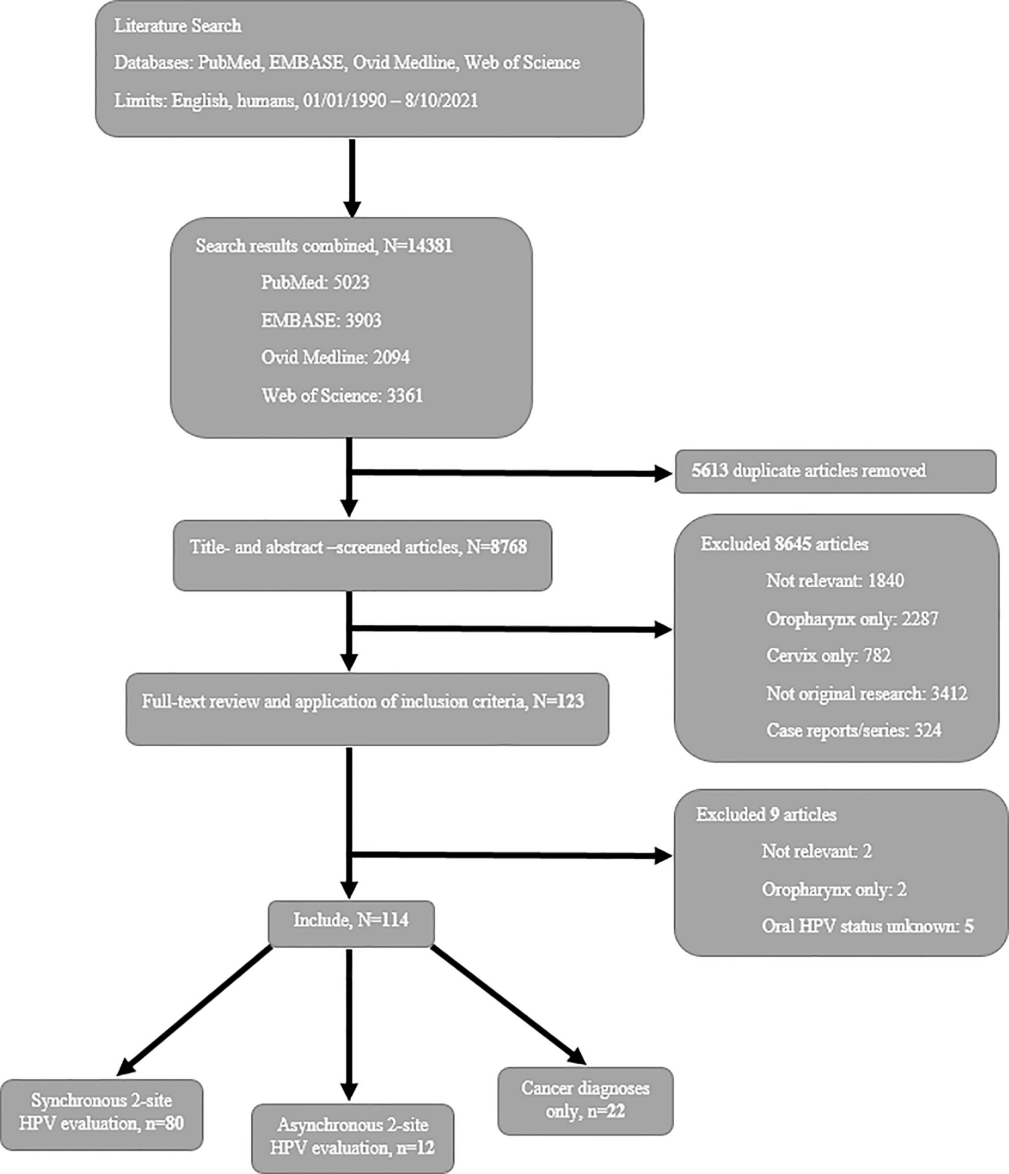
Flow diagram of study selection for a systematic review of oral-cervical HPV infection/cancer epidemiology literature.

A total of 8768 papers were identified through PubMed, EMBASE, Ovid Medline, and Web of Science databases after removing duplicates (**Figure 1**). Titles, abstracts, and full-text papers were screened, 8654 did not meet the eligibility criteria and subsequently were removed. Specifically, 1842 (21%) studies were not topic relevant, 3071 (35%) studies evaluated HPV only in the oropharynx/oral cavity [2289 (26%) studies] or cervix [782 (9%) studies], 5 (0.06%) studies did not relate oral cancers to HPV status, 3412 (39%) studies were not original research, and 324 (4%) studies were case reports or series. A total of 114 papers were included.

Most studies (n=80, 70%) (4, 15–93) evaluated oral and cervical sites with synchronous HPV testing; the remaining studies were divided between asynchronous evaluations (n=12, 11%) (94–105) and cancer diagnoses only (n=22, 19%) (106–127). Combining synchronous (4, 15–93) and asynchronous (94–105) study data, the overall estimate of oral and cervical dual-site HPV infections was 16% and the overall estimate of oral-cervical HPV type concordance among dually-infected women was 41% (**Table 1**). Among cancer diagnoses only studies (106–127), the incidence of a secondary cervical cancer diagnosis ranged from as few as 4.5/10,000 to as many as 192.5/10,000 women; the incidence of a secondary oral HPV-related cancer ranged from 1.0 to 45.8 per 10,000 women.

### Synchronous Oral-Cervical HPV Testing (n=80)

Eighty studies synchronously evaluated HPV-related infections at both the oral and cervical sites (4, 15–93) (**Table 2A**). Cervical samples were collected by a variety of measures with most studies using swabs or a combination of methods; oral samples were collected mainly by rinses or swabs. HPV DNA detection was most often determined through polymerase chain reaction (PCR) (n=60, 75%) (4, 16–20, 24–29, 33–35, 38, 39, 42–49, 52–56, 61–65, 67–76, 78–89, 91–93).

**Table 2A:** Description of methodology used in synchronous oral-cervical HPV evaluation papers (n=80).

AUTHOR, YEAR (REFERENCE)	STUDY DESIGN STUDY NAME	COUNTRY	PARTICIPANTS	SAMPLE COLLECTION METHOD	HUMAN PAPILLOMAVIRUS (HPV) DETECTION METHOD
				Cervical/Oral	
**SYNCHRONOUS**					
Papers with sufficient concurrent* data					
Mosmann et al., 2021 (84)	Cross-sectional	Argentina	n=100 women (n=50 abnormal cervical cytology; n=50 normal cervical cytology)	Cervical: Brush/swab Oral: Swab/scrape	Polymerase Chain Reaction (PCR)
Paaso et al., 2021 (85)	Cross-sectional Finnish Family HPV Study	Finland	n=21 women	Cervical: Brush Oral: Brush	PCR
Gilles et al., 2020 (86)	Cross-sectional	Belgium	n=44 human immunodeficiency virus (HIV)-positive women (n=22 women with perinatally infected HIV; n=22 with non-perinatal HIV)	Cervical: Smear Oral: Rinse	PCR
Nasioutziki et al., 2020 (87)	Cross-sectional	Greece	n=118 women with abnormal pap or high grade cervical intraepithelial neoplasia (CIN)	Cervical: Spatula and brush Oral: Rinse	PCR
Nemesio et al., 2020 (88)	Cross-sectional	Brazil	n=406 non-pregnant women with abnormal cervical cytology (n=323 with < CIN2; n=83 with ≥ CIN2)	Cervical: Cytology and colposcopy biopsy Oral: Rinse	PCR
Perez Quintanilla et al., 2020 (89)	Cross-sectional	Mexico	n=174 HIV+ women	Cervical: Brush Oral: Brush	PCR and reverse hybridization
Sricharoenchai et al., 2020 (90)	Cross-sectional	Thailand and Vietnam	n=192 HPV unvaccinated women (12-24 years old) (n=93 perinatally HIV+; n=99 HIV-)	Cervical: Vaginal speculum exam Oral: Rinse	Linear Array
Suehiro et al., 2020 (91)	Cross-sectional	Brazil	n=254 women (n=115 HIV+, n=139 HIV- women)	Cervical: Brush and spatula Oral: Brush and rinse	PCR and Multiplex Kit
Tewari, 2020 (93)	Cross-sectional	Ireland	n=235 women with abnormal cytology	Cervical: Biopsy Oral: Rinse	Cervical: Reverse hybridization Oral: PCR and reverse hybridization
Brouwer et al., 2019 (18)	Cross-sectional National Health and Nutrition Examination Survey (NHANES) 2003-2014	United States	n=10776 women with genital samples n=7102 women with oral samples	Cervical: Swab Oral: Rinse	PCR
Eggersmann et al., 2019 (17)	Cross-sectional	Germany	n=221 women	Cervical: Smear Oral: Smear and Rinse	PCR
Enerly 2019 (92)	Cross-sectional	Norway	n=312 girls (18-20 year olds) (n= 239 HPV vaccinated; n=73 HPV unvaccinated)	Cervical: Brush Oral: Swab	PCR and type specific hybridization
Kiwerska et al., 2019 (16)	Cross-sectional	Poland	n=197 women with previous history of cervical cancer or pre-cancerous lesions	Cervical: Brush Oral: Swab	PCR
Sehnal, 2019 (15)	Cross-sectional	Czech Republic	n=718 women (n=473 with high-grade cervical lesions; n=245 with low-grade/no cervical lesions)	Cervical: Brush Oral: Rinse	Linear Array
Cossellu et al., 2018 (25)	Cross-sectional	Italy	n=44 women with abnormal Pap smear (20-45 years old)	Cervical: Swab Oral: Swab	PCR
Grimm et al., 2018 (23)	Cross-sectional	Germany	n=73 women with cervical high grade squamous intraepithelial lesion (HSIL)	Cervical: Swab Oral: Swab	Linear Array and “PapilloCheck”
Thorsteinsson et al., 2018 (21)	Cross-sectional Study on HIV, cervical Abnormalities and infections in women in Denmark (SHADE) cohort	Denmark	n=214 HIV+ women	Cervical: Swab Oral: Swab	PCR
Tuominen et al., 2018 (20)	Cross-sectional Finnish Family HPV Study	Finland	n=39 women who recently gave birth	Cervical: Scrape Oral: Scrape	PCR
Vargas-Robles et al., 2018 (19)	Cross-sectional	Venezuela	n=111 women from the Amazonian area (12-53 years old)	Cervical: Swab Oral: Swab	PCR
Beachler et al., 2017 (73)	Cohort Costa Rica Vaccine Trial (CVT)	Costa Rica	n=352 women (22-33 years old)	Cervical: Not specified Oral: Rinse	PCR
Oliveira et al., 2017 (27)	Cross-sectional	Brazil	n=76 women	Cervical: Scrape Oral: Scrape/brush	PCR
Woelber et al., 2017 (26)	Cross-sectional	Germany	n=235 women with abnormal cervical cytology (18-45 years old)	Cervical: Swab Oral: Scrape	PCR
Kedarisetty et al., 2016 (32)	Cross-sectional NHANES 2009-2012	United States	n=3463 women	Cervical: Swab Oral: Rinse	Cervical: Linear Array and “Digene HC2 HPV DNA” test Oral: PCR
Kero et al., 2016 (74)	Cohort Finnish Family HPV Study	Finland	n=131 women	Cervical: Brush/scrape Oral: Brush/scrape	PCR
Loverro et al., 2016 (31)	Cross-sectional	Italy	n=35 transgendered individuals with cervix (14 female to male; 21 male to female)	Cervical: Brush Oral: Brush	Linear Array
Menezes et al., 2016 (75)	Cohort	India	n=65 women, HIV positive	Cervical: Swab Oral: Swab	PCR
Temizkan et al., 2016 (30)	Cross-sectional	Turkey	n=30 women with abnormal cervical cytology n=68 women with normal cervical cytology	Cervical: Smear Oral: Brush	None (cytology)
Uken et al., 2016 (29)	Cross-sectional	Germany	n=101 women with cervical dysplasia	Cervical: Brush Oral: Brush	PCR
Brouwer et al., 2015 (36)	Cross-sectional NHANES 2003-2012	United States	n=10407 women with genital samples n=5236 women with oral samples	Cervical: Swab Oral: Rinse	Cervical: Linear Array and multiplex assay Oral: PCR
Grun et al., 2015 (35)	Cross-sectional	Sweden	n=211 women (15-23 years old)	Cervical: Swab Oral: Swab	PCR
Skoczynski et al., 2015 (34)	Cross-sectional	Poland	n=152 pregnant women	Cervical: Smear Oral: Smear	PCR
Tatar et al., 2015 (33)	Cross-sectional	Hungary	n=149 women	Cervical: Not specified Oral: Rinse/brush	PCR
Ribeiro et al., 2014 (38)	Cross-sectional Recife Brazil Study	Brazil	n=31 women	Cervical: Swab/brush Oral: Swab/brush	PCR
Lima et al., 2014 (41)	Cross-sectional	Brazil	n=200 women (n=100 HIV positive, n=100 HIV negative)	Cervical: Brush Oral: Scraping/brush	“Digene HC2 HPV DNA” test
Louvanto et al., 2014 (76)	Case-control Finnish Family HPV Study	Finland	n=43 pregnant women with persistent cervical HPV infection (>24 months) n=52 pregnant women without cervical HPV infection	Cervical: Scrape/brush Oral: Scrape/brush	PCR
Mbulawa et al., 2014 (40)	Cross-sectional	South Africa	n=221 women (18-65 years old)	Cervical: Not specified Oral: Brush	Linear Array
Meyer et al., 2014 (39)	Cross-sectional	Germany	n=129 women	Cervical: Smear/brush Oral: Smear and rinse	PCR
Steinau et al., 2014 (37)	Cross-sectional NHANES	United States	n=1812 women (18-59 years old)	Cervical: Swab Oral: Rinse	Linear Array
Adamopoulou et al., 2013 (47)	Cross-sectional	Greece	n=43 women	Cervical: Scrape Oral: Rinse	PCR
Lang Kuhs et al., 2013 (44)	Cross-sectional CVT	Costa Rica	n=5838 women (22-29 years old)	Cervical: Exfoliated Oral: Rinse	PCR
Schlecht et al., 2013 (43)	Cross-sectional	United States	n=97 women (15-22 years old)	Cervical: Brush Oral: Rinse	PCR
Vogt et al., 2013 (42)	Cross-sectional	South Africa	n=34 women	Cervical: Swab Oral: Rinse	PCR
Du et al., 2012 (50)	Cross-sectional	Sweden	n=408 female youth (15-23 years old)	Cervical: Swab Oral: Rinse	Cervical: Multiplex array Oral: “Gentra Puregene Buccal Cell Kit”
Elasbali et al., 2012 (49)	Cross-sectional	Sudan	n=50 women (n=40/50 with cervical lesions; n=10/50 with no cervical lesions)	Cervical: Scrape Oral: Scrape	PCR
Zonta et al., 2012 (72)	Cross-sectional	Brazil	n=409 women inmates	Cervical: Brush Oral: Brush	PCR
Brown et al., 2011 (55)	Cross-sectional	Peru	n=184 female sex workers	Cervical: Not specified Oral: Rinse	PCR
Crawford et al., 2011 (54)	Cross-sectional	United Kingdom	n=100 women with abnormal cervical smear	Cervical: Swab/brush Oral: Swab	PCR
Matsushita et al., 2011 (52)	Cross-sectional	Japan	n=196 female sex workers (18-45 years old)	Cervical: Scrape/smear Oral: Scrape	PCR
Paaso et al., 2011 (77)	Cohort Finnish Family HPV Study	Finland	n=323 women	Cervical: Scrape Oral: Scrape	Multiplex Kit
Ragin et al., 2011 (51)	Cross-sectional Epidemiologic Study of HEalth Risk (ESTHER) project	United States	n=118 women	Cervical: Brush Oral: Brush and/or rinse	Linear Array
Termine et al., 2011 (4)	Cross-sectional	Italy	n=100 women with cervical HPV infection	Cervical: Not specified Oral: Exam, rinse	PCR
Termine et al., 2009 (56)	Cross-sectional	Italy	n=140 women with known cervical HPV status	Cervical: Spatula and brush Oral: Brush	PCR
Marais et al., 2008 (58)	Cross-sectional	South Africa	n=115 women with CIN1-CIN3	Cervical: Swab Oral: Swab	Linear Array
Richter et al., 2008 (57)	Cross-sectional	South Africa	n=30 women, HIV-positive (22-64 years old)	Cervical: Smear Oral: Brush	Linear Array
Nordin et al., 2007 (61)	Cross-sectional	(Not specified - Swedish author group)	n=30 women (n=21/30 renal transplant carriers; n=9/30 healthy controls)	Cervical: Brush Oral: Swab	PCR
Passmore et al., 2007 (60)	Cross-sectional	South Africa	n=103 women (18-40 years old)	Cervical: Swab Oral: Swab	Linear Array
Ragin et al., 2007 (59)	Cross-sectional	Tobago	n=212 women (18-65 years old)	Cervical: Brush Oral: Rinse	Linear Array; nested PCR
Fakhry et al., 2006 (70)	Cross-sectional Women’s Interagency HIV Study	United States	n=258 women (n=172 HIV positive, n=86 HIV negative)	Cervical: Rinse Oral: Rinse	PCR
Goncalves et al. 2006 (71)	Cross-sectional	Brazil	n=140 women (n=70/140 with clinical genital HPV lesions)	Cervical: Smear Oral: Swab	PCR
Canadas et al., 2004 (63)	Cross-sectional	Spain	n=188 women who practiced prostitution	Cervical: Not specified (exfoliates) Oral: Not specified	PCR
Smith et al., 2004 (62)	Cross-sectional	United States	n=577 pregnant women	Cervical: Swab Oral: Rinse	PCR
Scala et al., 2002 (64)	Cross-sectional	Italy	n=76 women treated for genital, anorectal, and perineal HPV lesions	Cervical: Scrape Oral: Not specified	PCR
Aaltonen et al., 2001 (67)	Cross-sectional	Finland	n=9 women with adult onset laryngeal papilloma patients	Cervical: Scraping Oral: Rinse	PCR
Marais et al., 2001 (65)	Cross-sectional	South Africa	n=81 women with CIN or squamous carcinoma of the cervix	Cervical: Swab/brush and biopsy Oral: Swab	PCR and enzyme-linked immunosorbent assay
Badaracco et al., 1998 (68)	Cross-sectional	Italy	n=29 women (21-48 years old)	Cervical: Spatula Oral: Swab	PCR
van Doornum et al., 1994 (78)	Cohort	Netherlands	n=162 women	Cervical: Spatula Oral: Spatula	PCR
van Doornum et al., 1992 (69)	Cross-sectional	Netherlands	n=111 women	Cervical: Spatula Oral: Spatula	PCR
Papers with insufficient concurrent* data					
Cranston et al., 2018 (24)	Cross-sectional within a randomized control trial AIDS Clinical Trials Group (ACTG)	United States and Brazil	n=103 HIV-infected women	Cervical: Swab Oral: Rinse	PCR
Sohn et al., 2018 (22)	Cross-sectional	Thailand, Vietnam	n=93 adolescents HIV positive n=99 adolescents HIV negative (12-24 years)	Cervical: Pap smear Oral: Rinse	Linear Array
Ciccarese et al., 2017 (28)	Cross-sectional	Italy	n=46 women	Cervical: Brush Oral: Brush	PCR
Kero et al., 2014 (79)	Cohort Finnish Family HPV Study	Finland	n=46 women	Cervical: Scrape/brush Oral: Brush	PCR
da Mota Vasconcelos Brasil et al., 2013 (46)	Cross-sectional	Brazil	n=31 women	Cervical: Brush Oral: Brush	PCR
Herrero et al., 2013 (45)	Cross-sectional CVT	Costa Rica	n=5840 women (18-25 years old)	Cervical: Collection of exfoliated cells Oral: Rinse	PCR
Schlecht et al., 2012 (48)	Cross-sectional	United States	n=645 young women (12-19 years old)	Cervical: Brush Oral: Rinse	PCR
Kero et al., 2011 (53)	Cross-sectional Finnish Family HPV Study	Finland	n=128 pregnant women (third trimester)	Cervical: Brush Oral: Brush	PCR
Sarkola et al., 2009 (80)	Cohort Finnish Family HPV Study	Finland	n=178 women (n=78 pregnant women, n=100 non-pregnant women)	Cervical: Scrape Oral: Scrape	PCR
D’Souza et al., 2007 (81)	Cohort Women’s Interagency HIV Study	United States	n=199 women (n=136 HIV positive, n=63 HIV negative)	Cervical: Rinse Oral: Rinse	PCR
Rintala et al., 2005 (82)	Cohort Finnish Family HPV Study	Finland	n=76 women	Cervical: Scrape/brush Oral: Scrape/brush	PCR
Winer et al., 2003 (83)	Cohort	United States	n=603 women, university students	Cervical: Swab Oral: Brush	PCR
Chatterjee et al., 2001 (66)	Cross-sectional	India	n=27 cervical samples from female prostitutes, n=69 oral samples from female prostitutes	Cervical: Smear Oral: Swab	Deoxyribonucleic acid hybridization (Vira type; Digene Diagnostics)

*Concurrent refers to the synchronous occurrence of any HPV type(s) in both the cervix and oral cavity/oropharynx.

**Table 2B:** Description of methodology used in asynchronous oral-cervical HPV evaluation papers (n=12).

AUTHOR, YEAR (REFERENCE)	STUDY DESIGN	COUNTRY	PARTICIPANTS	SAMPLE COLLECTION METHOD		HUMAN PAPILLOMAVIRUS (HPV) DETECTION METHOD	NOTE/“FOLLOW UP TIMING”
				Cervical/Oral sample type	Cervical intraepithelial neoplasia (CIN)- cervical cancer/Oral cancer diagnosis data source		
**ASYNCHRONOUS**							
Papers with sufficient dual-site** data							
Sanchez-Siles et al., 2020 (105)	Cohort	Spain	n=100 women (n=50 with HPV-related cervical intraepithelial neoplasia (CIN), n=50 without CIN)	Cervical: Not specified Oral: Rinse	Cervical: Hospital database Oral: N/A	PCR	Cervical: Not specified Oral: Baseline
Christensen et al., 2019 (104)	Case-control	Denmark	n=417 women with oropharyngeal squamous cell carcinoma	Cervical: N/A Oral: Tumor specimens	Cervical: Cancer registry Oral: Cancer registry	PCR	Cervical: Cancer history timing not specified, noted to be before oral cancer Oral: Cancer diagnosed 2000-2014
Rietbergen et al., 2018 (102)	Cross-sectional	Netherlands	n=308 women with invasive squamous cell carcinoma of the oropharynx	Cervical: N/A Oral: Biopsy	Cervical: Pap smears from pathology database Oral: Cancer registry	PCR	Cervical: Various years specified Oral: Treated 2000-2015
Lupato et al., 2017 (99)	Cross-sectional	Italy	n=253 women (18-35 years old)	Cervical: Self-report from Papanicolaou (Pap) smear Oral: Rinse	Cervical: N/A Oral: N/A	Not specified	Cervical: Current infections and Pap history (timing not specified) Oral: Baseline
Visalli et al., 2016 (100)	Cross-sectional	Italy	n=125 (n=100 women with pre-existing HPV genital lesions, n=25 healthy controls)	Cervical: Medical record Oral: Rinse	Cervical: N/A Oral: N/A	PCR	Cervical: Not specified Oral: Baseline
Marques et al., 2015 (101)	Cross-sectional	Brazil	n=43 women with CIN2, CIN3, and invasive cervical carcinoma	Cervical: N/A Oral: Brush	Cervical: Self-report from Pap Smear; Colposcopy Oral: N/A	PCR	Cervical: 1-6 months before baseline Oral: Baseline
Peixoto et al., 2011 (103)	Cross-sectional	Brazil	n=100 women with history of cervical HPV infection	Cervical: Histology Oral: Swab/scrape/brush/biopsy	Cervical: N/A Oral: N/A	PCR	Cervical: Not specified Oral: Baseline
Saini et al., 2010 (98)	Cross-sectional	Malaysia	n=70 women previously diagnosed with cervical cancer	Cervical: N/A Oral: Swab	Cervical: Medical records Oral: N/A	“Digene HC2 HPV DNA” test	Cervical: Not specified, undergoing active treatment Oral: Baseline
Sánchez-Vargas et al., 2010 (95)	Cross-sectional	Mexico	n=46 women with a CIN diagnosis <6 months	Cervical: N/A Oral: Swab	Cervical: Histology Oral: N/A	PCR	Cervical: <6 months from baseline Oral: Baseline
Premoli-De-Percoco, 1998 (97)	Cross-sectional	Not Specified	n=50 women with oral squamous cell carcinoma	Cervical: Swab Oral: Biopsy	Cervical: N/A Oral: Medical records	Non-radioactive DNA probes (Oligoprobe source, Polar Brewing Co.)	Cervical: Baseline Oral: Cancer history timing not specified
Kellokoski et al., 1992 (96)	Cross-sectional	Finland	n=334 women	Cervical: Biopsy Oral: Biopsy	Cervical: N/A Oral: N/A	Southern Blot Hybridization and PCR	Cervical: Medical histories starting in 1981 Oral: Baseline (no year, publication date 1992)
Papers with insufficient dual-site** data							
D’Souza et al., 2014 (94)	Cross-sectional	United States	n=104 women total (n=17/104 women were patients with HPV-positive oropharyngeal squamous cell cancer (OPC); n=87/104 were women partners of male patients with HPV-positive OPC)	Cervical: N/A Oral: Rinse	Cervical: Self-report, medical records Oral: Patients-previously diagnosed; partners-oral screening evaluation by oncologist	PCR	Cervical: Previous cancer history disclosed at study baseline without date Oral: Baseline

**Dual-site refers to the asynchronous occurrence of any HPV type(s) in both the cervix and oral cavity/oropharynx.

N/A: not applicable sample collection method, meaning a site specific sample was collected or a site specific cancer diagnosis was provided.

**Table 2C:** Description of methodology used in oral-cervical human papillomavirus (HPV)-related cancer diagnoses only papers (n=22).

AUTHOR, YEAR (REFERENCE)	STUDY DESIGN REGISTRY NAME	COUNTRY	PARTICIPANTS	PRIMARY CANCER DIAGNOSIS	DATA SOURCES	CANCER CONFIRMATION METHOD	TIME PERIOD
**CANCER DIAGNOSES ONLY**							
Holstead et al., 2020 (122)	Cohort	United States	n= 155 diagnosed with oropharyngeal squamous cell carcinomas (OPSCC) n=26 women diagnosed with HPV-positive OPSCC	Oral	Cancer registry (local) and medical records	Biopsy-proven	2012-2014
Larish et al., 2020 (123)	Cohort	United States	n=46 women diagnosed with HPV-positive OPSCC	Oral	Medical records	Not discussed HPV-positive: p16 staining or HPV DNA	N/A
Loopik et al., 2020 (124)	Cohort	The Netherlands	n=89018 women diagnosed with cervical intraepithelial neoplasia (CIN) 3 n=89018 women with a benign dermal nevus were selected as control group	Cervical (CIN3)	Histo and cytopathology registry (nationwide)	Histologically proven	1990-2010
Preti et al., 2020 (125)	Cohort	Italy	n=5595 patients surgically treated for high-grade CIN and had follow up times of at least 5 years	Cervical (High grade CIN)	Medical record with cancer registry (provincial)	Classification of Diseases (ICD) codes	1992-2014
Wang et al., 2020 (126)	Cohort Surveillance, Epidemiology, and End Results (SEER)	United States	n=63,710 women diagnosed with an index P-HPV-associated cancer	Oral or Cervical	Cervical: Cancer registry (state/nationwide) Oral: Cancer registry (state/nationwide)	ICD codes and histology codes	2000-2015
Gazzaz et al., 2019 (127)	Cohort Alberta Health	Canada	n=372 women diagnosed with OPSCC	Oral or Cervical	Cancer registry (provincial)	Not discussed	1997-2015
Papatla et al., 2019 (115)	Cohort SEER	United States	n=21060 women with cervical squamous cell carcinoma	Cervical	Cervical: Cancer registry (state/nationwide) Oral: Cancer registry (state/nationwide)	ICD codes	1973-2014
Suk et al., 2018 (117)	Cohort SEER	United States	n=44011 women with cervical cancer n=15303 women with oropharyngeal cancer	Oral or Cervical	Cervical: Cancer registry (state/nationwide) Oral: Cancer registry (state/nationwide)	ICD codes and histologically confirmed	1973-2014
Ebisch et al., 2017 (109)	Cohort PALGA	Netherlands	n=89018 women with CIN3	Cervical (CIN3)	Cervical: Cancer registry (nationwide) Oral: Cancer registry (nationwide)	Not discussed	1990-2010
Neumann et al., 2016 (114)	Cohort K2-France	France	n=6049 women with potentially-HPV-related first cancers (n=4234 cervical cancer; n=502 head and neck cancer)	Oral or Cervical	Cervical: Cancer registry (8 areas of France) Oral: Cancer registry (8 areas of France)	ICD codes	1989-2004
Svahn et al., 2016 (118)	Cohort Danish Cancer Registry	Denmark	n=101974 women with CIN3 (includes adenocarcinoma *in situ*)	Cervical (CIN3)	Cervical: Cancer registry (nationwide) Oral: Cancer registry (nationwide)	Pathology database matched	1943-2012
Jung et al., 2015 (113)	Cohort Korea Central Cancer Registry	South Korea	n=11322 women diagnosed with primary head and neck cancer	Oral	Cervical: Cancer registry (nationwide) Oral: Cancer registry (nationwide)	ICD codes	1993-2010
Gaudet et al., 2014 (110)	Cohort British Columbia (BC) Cancer Agency Cervical Cancer Screening Program	Canada	n=54320 women with CIN2 and CIN3	Cervical (CIN2, CIN3)	Cervical: Cancer registry (province-wide) Oral: Cancer registry (province-wide)	ICD codes	1980-2005
Skinner et al., 2014 (119)	Retrospective cohort University of Texas (UT) MD Anderson Cancer Center	United States	n=125 women with two or more HPV-related cancers	Oral or Cervical	Cervical: Institutional tumor registry (UT MD Anderson Cancer Center) Oral: Institutional tumor registry (UT MD Anderson Cancer Center)	Pathologically confirmed	1949-2009
Gan et al., 2013 (120)	Cohort	United States	n=2230 patients with confirmed squamous cell carcinoma of the oropharynx (SCCOP): oral cavity, oropharynx, hypopharynx, and/or larynx	Oral	Cervical: Medical chart review Oral: Medical chart review	Pathologically confirmed	1995-2010
Chen et al., 2012 (108)	Cohort Taiwan Cancer Registry	Taiwan	n=52972 women with cervical cancer	Cervical	Cervical: Cancer registry (nationwide) Oral: Cancer registry (nationwide)	ICD codes	1979-2008
Biron et al., 2011 (121)	Cohort Alberta Health Services	Canada	n=248 women with oropharyngeal squamous cell carcinoma	Oral	Cervical: Medical chart review Oral: Medical chart review	Pathologically confirmed	1998-2008
Chaturvedi et al., 2009 (107)	Cohort SEER	Denmark, Sweden, Norway, Finland, United States	n=104760 cervical cancers (n=85109 squamous cell carcinoma; n=10280 adenocarcinoma)	Cervical	Cervical: Cancer registry (multi-national combination) Oral: Cancer registry (multi-national combination)	Histology codes	1943-2002
Rose Ragin et al., 2008 (116)	Cohort SEER	United States	n=2618 women (19–97 years old) with cervical cancer	Cervical	Cervical: Cancer registry (state/nationwide) Oral: Cancer registry (state/nationwide)	ICD codes	1973-2002
Chaturvedi et al., 2007 (106)	Cohort SEER	Denmark, Sweden, Norway, Finland, United States	n=104760 women diagnosed with cervical cancer	Cervical	Cervical: Cancer registry (multi-national combination) Oral: Cancer registry (multi-national combination)	Not discussed	1943-2001
Hemminki et al., 2001 (112)	Cohort Swedish Family Cancer Database	Sweden	n=3366 women diagnosed with oral cancer n=17234 women diagnosed with cervical cancer	Oral or Cervical	Cervical: Cancer registry (nationwide) Oral: Cancer registry (nationwide)	Histologically or cytological confirmed	1958-1996
Hemminki et al., 2000 (111)	Cohort Swedish Family Cancer Database	Sweden	n=117830 women with *in-situ* cervical cancer n=17556 women with invasive cervical cancer	Cervical	Cervical: Cancer registry (nationwide) Oral: Cancer registry (nationwide)	ICD codes	1958-1996

Overall rates of cervical HPV+ and oral HPV+ cases varied by study (**Table 3A**). Almost all studies found higher rates of cervical HPV+ than oral HPV+ (n=76/80, 95%) (4, 15–19, 21–67, 69–79, 81–84, 86–93). On average, 53% of women were HPV+ in the cervix; an average of 15% of women were HPV+ in the oral cavity/oropharyngeal region. Most papers included high-risk HPV type results from DNA genotyping (n=74/80, 93%) (4, 15–29, 31–33, 35–37, 39–45, 47–66, 68–70, 72–93) with 82% (n=61/74) (4, 15–26, 29, 31, 33, 35–37, 40, 42, 44, 45, 47–52, 54–62, 65, 66, 69, 70, 72, 74–79, 81, 83–93) reporting exact HPV types observed.

**Table 3A:** Results of the synchronous oral-cervical HPV evaluation papers (n=80).

AUTHOR, YEAR (REFERENCE)	RESULTS, n=# of women unless otherwise noted				OVERALL QUALITY^§^
	Any Cervical Human Papillomavirus (HPV)+ or Oral HPV+ Infections	Concurrent^†^ Oral-cervical HPV Infections	Concordant^‡^ Oral-cervical HPV Infections	High Risk (HR)-HPV Infections	
**SYNCHRONOUS**					
**Papers with sufficient concurrent data**					
Mosmann et al., 2021 (84)	Cervical: n=18/100 (18%) (n=12 normal cervical cytology; n=6 abnormal cervical cytology) Oral: n=14/100 (14%) (n=9 normal cervical cytology; n=5 abnormal cervical cytology)	n=5/100 (5%) (n=3 normal cervical cytology; n=2 abnormal cervical cytology)	n=3/5* (60%)	Cervical: n=11/18* Oral: n=10/14*	Moderate
Paaso et al., 2021 (85)	Cervical: n=5/21 (24%) Oral: n=8/21 (38%)	n=2/21 (10%)	n=0/2 (0%)	Cervical: n=0/5* Oral: n=3/8*	Moderate
Gilles et al., 2020 (86)	Cervical: n=11/36 (31%), (n=6 perinatal human immunodeficiency virus (HIV); n=5 non-perinatal HIV) Oral: n=1/36 (3%), (n=1 perinatal HIV)	n=1/36 (3%), (n=1 perinatal HIV)	n=1/1 (100%)	Cervical: n=11/11* Oral: n=1/1*	Moderate
Nasioutziki et al., 2020 (87)	Cervical: 88/118 (75%) Oral: n=3/118 (3%)	Aggregate data only*	Aggregate data only*	Cervical= 74/118* Oral= 3/3*	Moderate
Nemesio et al., 2020 (88)	Cervical: n=251/401 (63%) Oral: n=16/406 (4%)	n=10/16 (63%)	n=9/10 (90%)	Cervical: n=251/251* Oral: n=16/16* (only HR HPV types tested)	Moderate
Perez Quintanilla et al., 2020 (89)	Cervical: n=168/174 (97%) Oral: n=161/174 (93%)	n=155/174 (89%)	n ≤ 39/155 (25%) Bar graph description*	Cervical: n=158/168* Oral: n=145/161*	Moderate
Sricharoenchai, 2020 (90)	Cervical: n=57/192 (30%) (n=34/93 perinatally HIV+; n=23/99 HIV-) Oral: n=8/192 (4%) (n=5/93 perinatally HIV+; n=3/99 HIV-)	Bar graph description (combined sites)	Bar graph description (combined sites)*	Cervical: n=57/192* Oral: n=8/192* (only HR HPV types tested)	Moderate
Suehiro et al., 2020 (91)	Cervical: n=103/254 (41%) (n=51/115 HIV+; n=53/139 HIV-) Oral: n=30/254 (12%), (n=17/115 HIV+; n=13/139 HIV-)	n=15/30 (50%) (n=8/17 HIV+; n=7/13 HIV-)	n=0/15 (0%)	Cervical: n=56/103* Oral: n=12/30*	Moderate
Tewari, 2020 (93)	Cervical: n=223/223 (100%) Oral: n=22/223 (10%)	n=21/22 (95%)	n=6/21* (29%)	Bar graph description*	Moderate
Brouwer et al., 2019 (18)	Cervical: n=2542/10776 (24%) Oral: n=282/7102 (4%)	Aggregate data only*	n=66	Bar graph description*	Moderate
Eggersmann et al., 2019 (17)	Cervical: n=144/221 (65%) Oral: n=1/221 (0.5%)	n=1/221 (0.5%)	.	Cervical: n=68/114* Oral: n=0/1	Moderate
Enerly et al. 2019 (92)	Cervical: n=122/312 (39%) (n=92/239 HPV vaccinated; n=30/73 HPV unvaccinated) Oral: n=4/312 (1.3%) (n=3/239 HPV vaccinated; n=1/73 HPV unvaccinated)	n=4/312 (1.3%)	n=2/4* (50%)	Cervical: n=60/312* (n=46/239 HPV vaccinated; n=14/73 HPV unvaccinated) Oral: n=1/312*	Weak
Kiwerska et al., 2019 (16)	Cervical: n=197/197 (100%) Oral: n=39/197 (20%)	n=39/197 (20%)	n=17/39 (44%)	Cervical: n=212/280 infections* Oral: n=30/52 infections*	Moderate
Sehnal et al., 2019 (15)	Cervical: n=448/714 (63%) Oral: n=10/438 (2%)	n=6/437 (1.4%)	n=5/6* (83%)	Cervical: n=416/448* Oral: n=10/10*	Moderate
Cossellu et al., 2018 (25)	Cervical: n=36/43 (84%) Oral: n=9/44 (20%)	n=7/44 (16%)	n=1/7* (14%)	Bar graph description*	Moderate
Grimm et al., 2018 (23)	Cervical: n=69/73 (95%) Oral: n=3/73 (4%)	n=3/73 (4%)	n=3/3* (100%)	Cervical: n=69/73* Oral: n=3/3*	Moderate
Thorsteinsson et al., 2018 (21)	Cervical: n=108/214 (50%) Oral: n=12/214 (6%)	n=0 (0%)	N/A	Cervical: n=60/108* Oral: n=8/12*	Moderate
Tuominen, 2018 (20)	Cervical: n=9/39 (23%) Oral: n=13/39 (33%)	n=4/39 (10%)	n=2/4* (50%)	Cervical: n=7/9* Oral: n=11/13*	Moderate
Vargas-Robles et al., 2018 (19)	Cervical: n=66/91 (73%) Oral: n=6/18 (33%)	Aggregate data only*	Aggregate data only*	Cervical: n=60/66* Oral: n=3/6*	Moderate
Beachler et al., 2017 (73)	Cervical: Year 4: n=223/350 (64%) infections (n=144/350 women, 41%); Year 6: n=40/223 (18%) infections Oral: Year 4: n=82/350 (23%) infections (n=66/350 women, 19%); Year 6: n=14/82 (17%) infections	Year 4: n=47/82 (57%) infections Year 6: n=3/47 (6%) infections	Year 4: n=31/47 (66%) infections Year 6: n=0/31 (0%) infections	Cervical: Year 4: n=131/223 infections; Year 6: n=26/131 infections Oral: Year 4: n=47/82 infections; Year 6: n=7/47 infections	Strong
Oliveira et al., 2017 (27)	Cervical: n=7/76 (9%) Oral: n=4/76 (5%)	n=1/76 (1%)	n=0/1 (0%)	Cervical: n=3/7 Oral: n=0/4	Weak
Woelber et al., 2017 (26)	Cervical: n=207/223 (93%) Oral: n=6/135 (4%)	n=6/135 (4%)	n=3/6* (50%)	Cervical: n=135/235* Oral: n=6/6*	Moderate
Kedarisetty et al., 2016 (32)	Cervical: n=1586/3463 (46%) Oral: n=141/3463 (4%)	n=107/3463 (3%)	n=41/107 (38%)	Cervical: n=337/1586 Oral: n=22/141	Moderate
Kero et al., 2016 (74)	Cervical: Baseline: n=25/131 (19.1%) 2 month (mo): n=14/105 (13%) 12 mo: n=51/114 (45%) 24 mo: n=60/101 (59%) 36 mo: n=56/101 (55%) 72 mo: n=10/45 (22%) Follow up: 13.3-59.4% Oral: Baseline: n=25/131 (19.1%) 2 mo: n=23/105 (22%) 12 mo: n=24/115 (21%) 24 mo: n=27/100 (27%) 36 mo: n=15/101 (15%) 72 mo: n=6/58 (10%) Follow up: 10.3-27.0%	Among 15 concordant couples: Baseline: n=2/15 (13%) 2 mo: n=0/15 (0%) 12 mo: n=2/15 (13%) 24 mo: n=5/15 (33%) 36 mo: n=1/15 (7%) 72 mo: n=1/15 (7%)	Among 15 concordant couples: Baseline: n=1/15* (7%) 2 mo: n=0/15 (0%) 12 mo: n=2/15* (13%) 24 mo: n=3/15* (20%) 36 mo: n=0/15 (0%) 72 mo: n=1/15* (7%)	Among 15 concordant couples: Cervical: Baseline: n=2/3* 2 mo: n=0/0 12 mo: n=6/8* 24 mo: n=9/10* 36 mo: n=7/7* 72 mo: n=3/3* Oral: Baseline: n=3/6* 2 mo: n=6/6* 6 mo: n=5/5* 12 mo: n=3/3* 24 mo: n=7/7* 36 mo: n=1/1* 72 mo: n=3/3*	Moderate
Loverro et al., 2016 (31)	Cervical: n=2/22 (9%) Oral: n=0/35 (0%)	n=0 (0%)	N/A	Cervical: n=1/2* Oral: N/A	Moderate
Menezes et al., 2016 (75)	Cervical: Baseline: n=26/50 (52%); Follow up: n=17/41 (41%) Oral: Follow up: n=5/38 (13%)	n=4 infections/38 women	.	Cervical: Baseline: n=24/50*; Follow up: n=16/41* Oral: Follow up: n=5/38*	Moderate
Temizkan et al., 2016 (30)	Cervical: n=30/98 (31%) Oral: n=3/98 (3%)	n=3/98 (3%)	.	.	Weak
Uken et al., 2016 (29)	Cervical: n=101/101 (100%) Oral: n=3/101 (3%)	n=3/101 (3%)	n=2/3* (67%)	Cervical: n=58/101* Oral: n=1/3*	Moderate
Brouwer et al., 2015 (36)	Cervical: n=1791/10407 (17%) Oral: n=196/5236 (4%)	n=116/3940 (3%)	n=45/116 (39%)	Bar graph description*	Moderate
Grun et al., 2015 (35)	Cervical: n=134/211 (64%) Oral: n=4/200 (2%)	n=4/200 (2%)	.	Cervical: Vaccinated: n=48/94; Not vaccinated: n=26/40* Oral: n=4/4*	Moderate
Skoczynski et al., 2015 (34)	Cervical: n=24/152 (16%) Oral: n=19/152 (13%)	n=14/152 (9%)	.	.	Moderate
Tatar, 2015 (33)	Cervical: n=33/40 (83%) Oral: n=8/40 (20%)	n=7/40 (18%)	n=5/7* (71%)	Cervical: n=25/33* Oral: n=4/8*	Moderate
Ribeiro et al., 2014 (38)	Cervical: n=18/31 (58%) Oral: n=17/31 (55%)	n=12/31 (38%)	n=7/12 (58%)	.	Moderate
Lima et al., 2014 (41)	Cervical: n=86/200 (43%) Oral: n=13/200 (7%)	n=6/200 (3%)	.	Cervical: n=77/86 Oral: n=9/86	Moderate
Louvanto et al., 2014 (76)	Cervical: Persistent cases: n=43/43 (100%); Controls: n=0/52 (0%) Oral: Persistent cases: n=13/43 (30%); Controls: n=11/51 (22%)	n=13/94 (14%) cases and controls	.	Cervical: n=43/43* Oral: Cases: n=13/13*	Strong
Mbulawa et al., 2014 (40)	Cervical: n=121/219 (55%) Oral: n=15/221 (7%)	Aggregate data provided	Aggregate data provided	Cervical: aggregate genital data provided* Oral: not separated by sex*	Moderate
Meyer et al., 2014 (39)	Cervical: n=70/129 (54%) Oral: n=7/129 (5%)	n=4/129 (3%)	n=1/4* (25%)	Cervical: n=94 infections/70 women Oral: n=3/7	Moderate
Steinau et al., 2014 (37)	n=1812 total Cervical: 42.7% population prevalence Oral: 3.8% population prevalence	3% population prevalence	6.4% same strain*	Bar graph description*	Moderate
Adamopoulou et al., 2013 (47)	Cervical: n=26/43 (60%) Oral: n=19/43 (44%)	n=18/43 (42%)	n= 15/18* (83%)	Cervical: n=17/26* Oral: n=14/19*	Moderate
Lang Kuhs et al., 2013 (44)	Cervical: n=1953/5838 (33%) Oral: n=101/5838 (2%)	n=35/5838 (0.6%)	.	Cervical: . Oral: n=57/101*	Moderate
Schlecht et al., 2013 (43)	Cervical: n=57/97 (59%) Oral: n=11/97 (11%)	n=8/97 (8%)	n=0/8 (0%)	Cervical: n=38/57 Oral: n=4/11	Moderate
Vogt et al., 2013 (42)	Cervical: n=31/34 (91%) Oral: n=4/34 (12%)	n=4/34 (12%)	n=2/4* (50%)	Cervical: n=25/31* Oral: n=1/4*	Moderate
Du et al., 2012 (50)	Cervical: n=129/174 (74%) Oral: n=37/401 (9%)	n=22/174 (13%)	n=20/22* (91%)	Cervical: n=113/129* Oral: n=20/24*	Weak
Elasbali et al., 2012 (49)	Cervical: n=40/50 (80%) Oral: n=1/50 (2%)	n=1/50 (2%)	.	Cervical: n=16/40* Oral: n=1/1*	Moderate
Zonta et al., 2012 (72)	Cervical: n=27/409 (7%) Oral: n=23/27 (85%)	n=18/27 (67%)	n=1/18* (6%)	Cervical: n=10/27* Oral: n=22/23*	Moderate
Brown et al., 2011 (55)	Cervical: n=121/184 (66%) Oral: n=14/184 (8%)	n=10/184 (5%)	.	Cervical: n=27/121* Oral: n=4/14*	Moderate
Crawford et al., 2011 (54)	Cervical: n=96/100 (96%) Oral: n=92/100 (92%)	n=88/100 (88%)	.	Cervical: n=198/245* infections Oral: n=197/226* infections	Moderate
Matsushita et al., 2011 (52)	Cervical: n=103/196 (53%) Oral: n=12/196 (6%)	n=6/196 (3%)	n=2/6* (33%)	Cervical: n=84/103* Oral: n=10/12*	Moderate
Paaso et al., 2011 (77)	Cervical: Baseline: n=54 infections/323 women 12-mo: n=106 infections/281 women 24-mo: n=146 infections/261 women 36-mo: n=138 infections/260 women Oral: n=0/316 (0%)	n=0 (0%)	N/A	Cervical: Baseline: n=42/54 infections* 12-mo: n=86/106 infections* 24-mo: n=132/146 infections* 36-mo: n=133/138 infections* Oral: N/A	Strong
Ragin et al., 2011 (51)	Cervical: n=37/110 (34%) Oral: n=12/118 (10%)	n=5/110 (5%)	n=1/5* (20%)	Cervical: n=20/37* Oral: n=5/12*	Weak
Termine et al., 2011 (4)	Cervical: n=98/98 (100%) Oral: n=14/98 (14%)	n=14/98 (14%)	n=3/14 (21%)	Among concurrent cases: Cervical: n=10/14* Oral: n=3/14*	Moderate
Termine et al., 2009 (56)	Cervical: n=76/140 (54%) Oral: n=2/140 (1%)	n=2/140 (1%)	n=0/2* (0%)	Cervical: n=38 infections/76 women* Oral: n=2/2*	Moderate
Marais et al., 2008 (58)	Cervical: n=98/109 (90%) Oral: n=28/105 (27%)	n=25/99 (25%)	n=5/25 (20%) (detected by sequencing, not linear array)	Cervical: n=190/216* infections Oral: n=10/33* infections	Moderate
Richter et al., 2008 (57)	Cervical: n=29/30 (97%) Oral: n=6/30 (20%)	n=6/30 (20%)	n=3/6* (50%)	Cervical: n=13/16* Oral: n=2/6*	Moderate
Nordin et al., 2007 (61)	Cervical: n=2/30 (7%) Oral: n=0/30 (0%)	n=0 (0%)	N/A	Cervical: n=1/2* Oral: N/A	Weak
Passmore et al., 2007 (60)	Cervical: n=92/103 (89%) Oral: n=22/91 (24%)	n=4/91 (4%)	n=4/4* (100%)	Cervical: n=68 infections/92 women* Oral: n=4 infections/22 women*	Moderate
Ragin et al., 2007 (59)	Cervical: n=75/212 (35%) Oral: n=14/212 (7%)	n=7/212 (3%)	n=1/7* (14%)	Cervical: n=43/75* Oral: n=3/14*	Weak
Fakhry et al., 2006 (70)	Cervical: n=479 infections/234 women Oral: n=69 infections/241 women	n=37/221 (17%)	n=14/37* (38%)	Cervical: n=224/479* infections Oral: n=30/69* infections	Moderate
Goncalves et al., 2006 (71)	Cervical: n=70/140 (50%) Oral: n=29/140 (21%)	n=26/140 (19%)	.	.	Moderate
Canadas et al., 2004 (63)	Cervical: n=52/187 (28%) Oral: n=15/188 (8%)	n=7/188 (4%)	n=3/7* (43%)	Cervical: n=41/65 infections Oral: n=4/15 infections	Moderate
Smith et al., 2004 (62)	Cervical: n=165/577 (29%) Oral: n=14/577 (2%)	n=6/577 (1%)	n=0/6 (0%)	Cervical: n=104/577* Oral: n=9/577*	Moderate
Scala et al., 2002 (64)	Cervical: n=22/76 (29%) Oral: n=2/76 (3%)	n=2/76 (3%)	.	n=8/10 (not separated by site)	Moderate
Aaltonen et al., 2001 (67)	Cervical: n=5/9 (55%) Oral: n=0/9 (0%)	n=0 (0%)	N/A	.	Moderate
Marais et al., 2001 (65)	Cervical: n=81/81 (100%) Oral: n=2/28 (7%)	n=2/28 (7%)	n=0/2 (0%)	Cervical: n=35/81* Oral: n=2/28*	Moderate
Badaracco et al., 1998 (68)	Cervical: n=8/24 (33%) Oral: n=11/29 (38%)	n=4/24 (17%)	n=3/4* (75%)	Cervical: n=6/8 Oral: n=7/11	Moderate
van Doornum et al., 1994 (78)	Cervical: Baseline: n=25/162 (15%); Follow up: n=59/99 infections (60%) in 110 women Oral: Baseline: n=0/162 (0%); Follow up: n=1/110 (1%)	n=0 (0%)	N/A	Cervical: Baseline: n=22/25* Oral: Follow up: n=1/1*	Moderate
van Doornum et al., 1992 (69)	Cervical: n=15/111 (14%) Oral: n=0/111 (0%)	n=0 (0%)	N/A	Cervical: n=12/15* Oral: N/A	Moderate
**Papers with insufficient concurrent data**					
Cranston et al., 2018 (24)	Cervical: n=65/103 (63%) Oral: n=115/575 (includes males and females) (20%)	.	Aggregate data only	Cervical: n=42/65* Oral: n=109 infections/115 people* (includes males and females)	Moderate
Sohn et al., 2018 (22)	Cervical: n=98/192 (51%) Oral: n=18/190 (9%)	.	.	Cervical: n=69/98* Oral: n=9/18*	Moderate
Ciccarese et al., 2017 (28)	Cervical: n=31/46 (67%) Oral: n=17/46 (37%)	.	.	Cervical: n=12/31 Oral: n=3/17	Moderate
Kero et al., 2014 (79)	Cervical: Baseline: n=8/46 (17%), Follow up: n=10/46 (22%) Oral: Baseline: n=3/46 (7%), Follow up: n=4/41 (10%)	.	.	Cervical: Baseline: n=0/8*, Follow up: n=9/10* Oral: Baseline: n=3/3*, Follow up: n=3/4*	Strong
da Mota Vasconcelos Brasil et al., 2013 (46)	Cervical: n=18/31 (58%) Oral: n=17/31 (55%)	.	.	.	Moderate
Herrero et al., 2013 (45)	Cervical: Baseline: n=511/5832 (9%); Year 4: n=280/5834 (5%) Oral: Year 4: n=157/2924 (5%)	.	.	Cervical: Year 4: n=280/5843* Oral: Year 4: n=57/5834	Moderate
Schlecht et al., 2012 (48)	Cervical: n=345/645 (53%) Oral: n=126/645 (20%)	.	.	Cervical: n=208 infections/345 women* Oral: n=17 infections/126 women*	Moderate
Kero et al., 2011 (53)	Cervical: n=24/128 (19%) Oral: n=22/128 (17%)	.	.	Cervical: n=19/24 Oral: n=18/22	Moderate
Sarkola et al., 2009 (80)	Cervical: Baseline: n=31/178 (17%) 36 mo: n=24/178 (13%) (Aggregate data and bar graph description provided for additional follow-up mo) Oral: Baseline: n=33/178 (19%) 6mo: n=43/178 (24%) (Aggregate data bar graph description provided for additional follow-up mo)	.	.	Cervical: Baseline: n=31/178 36 mo: n=24/178 (Aggregate data and bar graph description provided for additional follow-up mo) Oral: Baseline: n=33/178 6mo: n=43/178 (Aggregate data and bar graph provided for additional follow-up mo)	Strong
D’Souza et al., 2007 (81)	Cervical: Baseline: n=116/182 (64%); Follow up: n=110/182 (60%) Oral: Baseline n=35/182 (19%); Follow up: n=36/182 (20%)	.	.	Cervical: Baseline: n=82/116*; Follow up: n=90/110* Oral: Baseline n=21/35*; Follow up: n=21/36*	Strong
Rintala et al., 2005 (82)	Cervical: n=10/76 (13%) to n=19/76 (25%) Oral: n=6/76 (8%) to n=26/76 (34%) (Ranges presented; data aggregately reported for baseline to 24-mo follow-up)	.	.	Cervical: n=10/76 (13%) to n=19/76 (25%) Oral: n=6/76 (8%) to n=26/76 (34%) (Ranges presented; bar graph description and aggregate data reported for baseline - 24-mo follow-up)	Moderate
Winer et al., 2003 (83)	Cervical: Incident cases: n=88/444 (20%) Oral: n=5/2619 samples (0.2%) from 529 women	.	.	Aggregate data and bar graph description*	Moderate
Chatterjee et al., 2001 (66)	Cervical: n=17/27 (63%) Oral: n=20/69 (29%)	.	.	Cervical: n=17/17* Oral: n=20/20*	Moderate

N/A, Not applicable; ., No information provided; *HPV types listed in paper; **^†^**:Concurrent refers to the synchronous dual-site occurrence of any HPV type(s) in both the cervix and oral cavity/oropharynx; **^‡^** Concordant refers to infections with at least one identical HPV type across sites, synchronously or asynchronously; **^¶^**:HR-HPV types included 16, 18, 31, 33, 34, 35, 39, 45, 51, 52, 56, 58, 59, 66, 68, and 70; ^§^: Study quality assessed though the Quality Assessment Tool for Quantitative Studies (QATQS) from the Effective Public Health Practice Project.

**Table 3B:** Results of the asynchronous oral-cervical HPV evaluation papers (n=12).

AUTHOR, YEAR (REFERENCE)		RESULTS, n=# of women unless otherwise noted							OVERALL QUALITY^§^
		Any Cervical Human Papillomavirus (HPV)+ or Oral HPV+ Infections		Dual-site^††^ oral-cervical HPV infections		Concordant^‡^ dual-site oral-cervical infections		High Risk (HR)-HPV Infections	
**ASYNCHRONOUS**									
**Papers with sufficient dual-site data**									
Sanchez-Siles et al., 2020 (105)		Cervical: n=50/100 (50%) Oral: n= 13/100 (13%), (n=7/50 with cervical intraepithelial neoplasia (CIN), n=6/50 without CIN)		n=7/50 (14%)		n=1/50 (2%)		Cervical: n=62/93* infections Oral: n=12/16* infections	Strong
Christensen et al., 2019 (104)		Cervical: n=72/343 (21%) Oral: n=203/417 (49%)		n=42/343 (12%)		.		.	Strong
Rietbergen et al., 2018 (102)		Cervical: n=16/224 (7%) Oral: n=70/308 (23%)		n=9/224 (4%)		.		.	Moderate
Lupato et al., 2017 (99)		Cervical: n=11/90 (12%) Oral: n=10/253 (4%)		n=1/90 (1.1%)		.		Cervical: . Oral: HR-HPV cases not separated by sex*	Moderate
Visalli et al., 2016 (100)		Cervical: n=100/125 (80%) Oral: n=26/125 (21%)		n=24/125 (19%)		.		Cervical: n=58/100* Oral: bar graph description *	Moderate
Marques et al., 2015 (101)		Cervical: n=43/43 (100%) Oral: n=1/43 (2%)		n=1/43 (2%)		.		Cervical: . Oral: n=0/1	Moderate
Peixoto et al., 2011 (103)		Cervical: n=100/100 (100%) Oral: n=81/100 (81%)		n=81/100 (81%)		.		.	Moderate
Saini et al., 2010 (98)		Cervical: n=70/70 (100%) Oral: n=4/70 (6%)		n=4/70 (6%)		.		Cervical: . Oral: n=4/4	Moderate
Sánchez-Vargas et al., 2010 (95)		Cervical: n=43/43 (100%) Oral: n=43/43 (100%)		n=43/43 (100%)		.		Cervical: . Oral: n=15/43*	Moderate
Premoli-De-Percoco et al., 1998 (97)		Cervical: n=28/50 (56%) Oral: n=35/50 (70%)		n=23/50 (46%)		n=23/23* (100%)		Cervical: n=28/28* Oral: n=35/35*	Moderate
Kellokoski et al., 1992 (96)		Cervical: n=14/272 (5%) Oral: Southern Blot Hybridization (SBH): n=42/272 (15%); Polymerase chain reaction (PCR): n=25/85 (29%)		n=14/272 (5%)		n=2/14* (14%)		Controls: Cervical: n=12/25* Oral: n=6/25*	Moderate
**Papers with insufficient dual-site data**									
D’Souza et al., 2014 (94)		Cervical: n=11/104 (11%) Oral: n=13/104 (13%)		.		.		Cervical: . Oral: n=11/104	Moderate

.: No information provided; *:HPV types listed in paper; **^††^** Dual-site refers to the asynchronous occurrence of any HPV type(s) in both the cervix and oral cavity/oropharynx; **^‡^** Concordant refers to infections with at least one identical HPV type across sites, synchronously or asynchronously; **^¶^** HR-HPV types included 16, 18, 31, 33, 34, 35, 39, 45, 51, 52, 56, 58, 59, 66, 68, and 70; ^§^: Study quality assessed though the Quality Assessment Tool for Quantitative Studies (QATAS) from the Effective Public Health Practice Project.

**Table 3C:** Results of the oral-cervical human papillomavirus (HPV)-related cancer diagnoses only papers (n=22).

AUTHOR, YEAR (REFERENCE)	RESULTS, number of cancer diagnoses				OVERALL QUALITY^§^
	Primary Diagnosis: Cervical	Primary Diagnosis: Oral	Secondary Diagnosis: Cervical	Secondary Diagnosis: Oral	
**CANCER DIAGNOSES ONLY**					
Holstead et al., 2020 (122)		26	Cervical cancer: n=2		Strong
Larish et al., 2020 (123)		46	Cervical cancer: n=1 Cervical intraepithelial neoplasia (CIN) 1-3: n=12 High risk HPV+: n=5		Strong
Loopik et al., 2020 (124)	1797			Oropharyngeal cancers: n=0	Strong
Preti et al., 2020 (125)	3184			Oropharynx: n=5 Esophagus: n=1	Strong
Wang et al., 2020 (126)	46,550	6,288	Among women who had potentially HPV-associated cancer: n=2,488 had secondary cervical cancer Standardized incidence ratio (SIR)=1.50 (1.44-1.56) Among women who had oral cancer: n=5 had secondary cervical cancer, SIR=1.53 (0.49-3.56)	Among women who had potential HPV-associated cancer: n=695 had secondary oral cancer, SIR=2.29 (2.12-2.47) Among women who had cervical cancer: n=3 had secondary oral cancer SIR=3.88 (0.78-11.33)	Strong
Gazzaz et al., 2019 (127)		372	History of cervical cancer (CC): n=33 SIR of CC: Age 25-39: 12.8 Age 40-54: 108.9 Age 55-69: 77.7 Age 70+: 23.9		Strong
Papatla et al., 2019 (115)	21,060			Oropharynx: n=4 Oral cavity and pharynx: n=72 SIR: 4.36 (95% confidence interval (CI)=1.19-11.15)	Strong
Suk et al., 2018 (117)	44,011	15,303	Primary Oropharyngeal cancer: Cervical: n=17 SIR: 1.6, P<0.05	Primary cervical cancer: Oropharyngeal: n=56 SIR: 1.4, P<0.05	Moderate
Ebisch et al., 2017 (109)	89,018			Oropharyngeal: n=13 SIR: 5.51 (95% CI=1.22-24.84)	Strong
Neumann et al., 2016 (114)	4234	502	Among primary head and neck: Cervical: n=0/502	Among primary cervical: head and neck: n=5/4234 SIR: 6.34 (95% CI=2.04-14.79) tongue and tonsil: n=0/4234 oral cavity: n=0/4234 larynx: n=4/4234 SIR: 8.85 (95% CI=2.38-22.65)	Strong
Svahn et al., 2016 (118)	101,974			Any Head and Neck Squamous Cell Carcinoma: n=189 (Strongly HPV associated: n=63/189; Base of tongue and tonsil: n=47/63; Other oropharynx: n=16/63) Hazard ratio (HR): 1.99 (95% CI=1.72-2.31)	Strong
Jung et al., 2015 (113)		11,322	Among those with primary oral cavity: Cervical: 3 years (yrs): n=36; 5 yrs: n=73 SIR:0.55 (95% CI=0.11-1.6) Among those with primary oropharynx: Cervical: 3 yrs: n=97; 5 yrs: n=133 SIR: 3.11 (95% CI=1.14-6.77) Among those with primary larynx: Cervical: 3 yrs: n=0; 5 yrs: n=12 SIR: 0.47 (95% CI=0.01-2.63)		Strong
Gaudet et al., 2014 (110)	54,320			Head and neck: n=30 SIR: 0.61 (95% CI=0.21-1.38)	Strong
Skinner et al., 2014 (119)	85	17	Among those with primary head and neck cancer: Cervical: n=8 [NOTE: n=5 synchronous cancers at the cervix and head and neck sites]	Among those with primary cervical cancer: Head and neck: n=63	Moderate
Gan et al., 2013 (120)		2,230	Among those who had primary oropharynx cancer: Cervical: n=0 Among those who had primary non-oropharynx cancer: Cervical: n=1		Moderate
Chen et al., 2012 (108)	52,972			Oral/pharynx: n=37; SIR: 1.18 (95% CI=0.83-1.62) Salivary gland: n=4; SIR: 0.77 (95% CI=0.21-1.97) Nasopharynx and nasal cavity: n=34; SIR: 1.01 (95% CI=0.70-1.42) Esophagus: n=31; SIR: 2.55 (95% CI=1.74-3.63) Larynx: n=2; SIR: 0.67 (95% CI=0.08-2.43)	Strong
Biron et al., 2011 (121)		248	Cervical: n=20 SIR: 29.4 (95% CI=12.05-74.98)		Strong
Chaturvedi et al., 2009 (107)	104,760			HPV-related cancer: n=1248 Tongue: n=28; SIR: 1.25 (95% CI=0.83-1.81) Mouth: n=60; SIR: 1.61 (95% CI=1.24-2.08) Pharynx: n=49; SIR: 2.06 (95% CI=1.53-2.73) Esophagus; n=89; SIR: 1.50 (95% CI=1.21-1.86) Larynx: n=48; SIR: 2.10 (95% CI=1.55-2.79)	Strong
Rose Ragin et al., 2008 (116)	2,618			Oral Cavity: n=30 Oral cavity and pharynx SIR=1.7 (95% CI=1.3–2.2) Lip: n=4 Salivary gland: n=5 Oropharynx: n=12 (11 tonsil);	Strong
				SIR=2.7 (95% CI=1.4–4.7) Tonsils SIR=3.1(95% CI=1.5–5.5) Nasopharynx: n=2 Larynx: n=25; SIR=2.7 (95% CI=1.7–3.9) Hypopharynx: n=7	
Chaturvedi et al., 2007 (106)	104,760			Tongue: n=32; SIR: 1.18 (95% CI=0.81-1.67) Mouth: n=66; SIR: 1.48 (95% CI=1.15-1.89) Pharynx: n=52; SIR: 1.83 (95% CI=1.37-2.41) Esophagus: n=101; SIR: 1.42 (95% CI=1.16-1.73) Larynx: n=56; SIR 2.02 (95% CI=1.53-2.63)	Strong
Hemminki et al., 2001 (112)	17,234	3,366	Among those who had primary oral cancer: Cervical cancer: n=8 SIR: 1.73 (95% CI=0.74-3.13)	Among those who had primary cervical cancer: Oral: n=33 SIR: 2.20 (95% CI=1.51-3.01) Esophagus: n=9 SIR: 1.67 (95% CI=0.76-2.94)	Moderate
Hemminki et al., 2000 (111)	117,830	17,556		Among primary *in situ* cervical: n=101 upper aerodigestive tract SIR=1.68 Among primary invasive cervical cancer: n=31 upper aerodigestive tract SIR=2.45	Strong

^§^Study quality assessed though the Quality Assessment Tool for Quantitative Studies (QATAS) from the Effective Public Health Practice Project.

Eighty-three percent of all synchronous testing studies (n=67/80, **Table 3A**) (4, 15–21, 23, 25–27, 29–44, 47, 49–52, 54–65, 67–78, 84–93) provided some form of data on concurrent oral-cervical HPV+ cases. Concurrent oral and cervical HPV infection rates could be calculated for most, but not all, of these studies (n=59/67, 88%) (4, 15–17, 20, 21, 23, 25–27, 29–39, 41–44, 47, 49–52, 54–65, 67–72, 76–78, 84–89, 91–93). The calculated concurrent rates ranged from 0% to 95%, depending on the study. On average, 15% of women had HPV infections occurring concurrently in both sites. Most rates of concurrent oral and cervical HPV infections were ≤10% (n=39/59, 66%) (15, 17, 20, 21, 23, 26, 27, 29–32, 34–37, 39, 41, 43, 44, 49, 51, 52, 55, 56, 59–65, 67, 69, 77, 78, 84–86, 92). Only four studies (7%) (54, 72, 89, 93) had concurrent oral and cervical HPV infection rates over 65%.

Among the 67 studies identifying concurrent oral and cervical HPV+ cases, 70% (n=47/67) (4, 15, 16, 18–20, 23, 25–27, 29, 32, 33, 36–40, 42, 43, 47, 50–52, 56–60, 62, 63, 65, 68, 70, 72–74, 84–93) determined concordance in oral-cervical HPV types (**Table 3A**). For studies reporting overall rates (n=40/47, 85%) (4, 15, 16, 20, 23, 25–27, 29, 32, 33, 36–39, 42, 43, 47, 50–52, 56–60, 62, 63, 65, 68, 70, 72, 84–86, 88, 89, 91–93), concordance in oral and cervical HPV infection types ranged from 0% to 100%, with an average of 41% of the women having infections of the same type in both sites. More than half of the studies had oral-cervical HPV type concordance rates of <50% (n=23/40, 58%) (4, 16, 25, 27, 32, 36, 37, 39, 43, 51, 52, 56, 58, 59, 62, 63, 65, 70, 72, 85, 89, 91, 93), yet seven studies reported concordance rates of >80% (15, 23, 47, 50, 60, 86, 88).

### Asynchronous Oral-Cervical HPV Testing (n=12)

Twelve studies evaluated HPV-related infections of the oral cavity/oropharynx and cervix asynchronously (94–105) (**Table 2B**). Most studies sampled women with cervical infections for oral HPV (n=7/12, 58%) (95, 96, 98, 100, 101, 103, 105). Cervical HPV data collection usually relied on medical records (94–96, 98, 100–105) while at least some oral samples were actively evaluated for HPV during the study (94–105). Oral HPV sampling methodology used a buccal (brush) sample (95, 98, 101, 103), biopsied lesions (96, 97, 102, 104), or a gargle/rinse sample (94, 99, 100, 105). Cervical and oral HPV DNA was often detected by PCR (94–96, 100–105).

Half of the asynchronous studies (n=6/12, 50%) showed that more women were HPV+ in the cervix than in the oral cavity/oropharynx (98–101, 103, 105) while essentially the other half (n=5/12, 42%) found the opposite (94, 96, 97, 102, 104). Most asynchronous studies (n=9/12, 75%) provided some data regarding the high-risk HPV types (94–101, 105) (**Table 3B**), tending to only specify when high-risk oral HPV was found (n=5/9, 56%) (94, 95, 98, 99, 101). Due to HPV assessments occurring at different times, studies rarely (n=4/9, 44%) reported both the specific high-risk oral and cervical HPV types found at the person level (96, 97, 100, 105).

Most asynchronous studies (n=11/12, 92%) differentiated between women with and without dual-site oral and cervical HPV infections at any time (95–105) (**Table 3B**). One woman (2%) to as many as all (100%) women asynchronously tested positive for HPV in both the oral cavity/oropharynx and cervix. The overall dual-site oral and cervical HPV+ infection rate estimate was 26% (95–105). On average, when women had (pre)existing cervical infections (95, 98, 100, 101, 103, 105), almost twice as many were dually-infected with HPV in the oral cavity/oropharynx (avg.: 37%, range: 2-100%) as compared to women with (pre)existing oral HPV infections who were also cervical HPV+ (avg.: 21%, range: 4-46%) (97, 102, 104). Women without a known, prior oral or cervical HPV infection were not as likely to be dually HPV infected at both sites, with rates ranging from 1.1-5% (96, 99).

Among studies where women were known to be dually-infected with oral and cervical HPV, 27% (n=3/11) measured concordance in HPV types across both sites at any time (96, 97, 105). On average, 39% of asynchronous oral and cervical infections within women had an HPV type in common (96, 97, 105) (**Table 3B**). Women who had an HPV+ oral cancer and a cervical HPV infection present had the greatest concordance in oral-cervical HPV types (100%) (96, 97, 105). Rates of concordant oral-cervical HPV types were lower in studies where not all women had prior HPV-related infections (2-14%) (96, 97, 105).

### Cancer Diagnoses Only (Primary Oral/Cervical, Secondary Cervical/Oral, n=22)

Twenty-two retrospective studies focused on the diagnosis of a secondary cervical or oral cancer after a primary cancer diagnosis of oral or cervical cancer (106–127) (**Table 2C**). Although we specifically included studies focused on HPV-related oral cancers, the sites of oral cancers varied across studies (e.g., some studies included oropharynx, oral cavity and pharynx, some only included oropharyngeal, and some vaguely defined HPV-related head and neck sites). Five studies examined the risk of a secondary cervical cancer after a primary diagnosis of oral cancer (113, 120–123). Half of the studies (n=11/22, 50%) examined the risk of a secondary oral cancer diagnosis after a primary diagnosis of a cervical cancer (n=6) (106–108, 111, 115, 116) or a cervical intraepithelial neoplasia (CIN) (n=5) (109, 110, 118, 124, 125). Six studies investigated the risk of a secondary cervical and/or oral cancer after a primary diagnosis of an oral and/or cervical cancer (112, 114, 117, 119, 126, 127). Most studies utilized data from country or state level cancer registries to monitor disease surveillance (n=15/22, 68%) (106–118, 124, 126); three studies conducted medical chart reviews (120, 121, 123); four studies collected at least some data from institutional or provincial tumor registries (119, 122, 125, 127).

Among women with a primary diagnosis of oral cancer, the number of secondary cervical cancers was lowest among medical record-based studies (122, 123), followed by provincial registries (121, 127), and highest among national studies (112, 113, 117, 120, 126). National studies reported that the incidence of a secondary cervical cancer ranged from 4.5-192.5 per 10,000 women (112, 113, 117, 120, 126) (**Table 3C**). The observed cases of a secondary cervical cancer were higher than expected in five studies with the SIR generally ranging from 1.4-29.4 (113, 117, 120, 121, 127). Interestingly, Gan et al. found that the SIR of a secondary cervical cancer was smaller among women diagnosed with HPV-related oral cancers (SIR range: 3.3-4.0) compared to women diagnosed with non-HPV-related oral cancers (SIR range: 8.3-12.8) (120). Two studies did not observe any differences between the numbers of observed and expected cases of a secondary cervical cancer among women who had a primary oral cancer (112, 126).

Among women with a primary cervical cancer, one provincial-level registry found very few cases of secondary oral cancers (125). Nationally, studies that reported the incidence of a secondary oral/head and neck cancer ranged from 1.0-45.8 per 10,000 women (106–108, 110–112, 114–117, 126); one study had an incidence of zero for secondary oropharyngeal cancers (124) (**Table 3C**). The incidence rates varied due to differences in included oral cancer sites across studies. The observed cases of a secondary oral cancer were higher than expected in almost all national studies, including primary CIN3 cases, with the SIR ranging from 1.4-6.3 (106–109, 111, 112, 114–118, 126).

### Quality Assessment

For the quality assessment of the 114 included papers based on the QATQS tool, 26 studies (23%) were classified as strong (73, 76, 77, 79–81, 104–111, 113–116, 118, 121–127), 81 studies (71%) were moderate (4, 15–26, 28, 29, 31–49, 52–58, 60, 62–72, 74, 75, 78, 82–91, 93–103, 112, 117, 119, 120), and 7 (6%) were weak (27, 30, 50, 51, 59, 61, 92) (**Tables 3A–C**). The most common component rated as weak was study design (n=79, 69%) (4, 15–72, 84–103); only a few studies used a case-control design (n=2, 2%) (76, 104) or cohort design (n=33, 29%) (73–75, 77–83, 105–127) with the majority being cross-sectional designs (n=79, 69%) (4, 15–72, 84–103). In addition, few studies randomly selected participants for inclusion from a comprehensive list of the target population (n=26, 23%) (18, 21, 24, 32, 34, 36, 37, 44, 45, 83, 92, 104, 108, 109, 111–113, 115–119, 121, 124, 126, 127). This contributed to most studies being classified as ‘moderate’ for selection bias (n=99, 87%) (4, 15–26, 28–33, 35–43, 46–49, 51–58, 60–77, 79–82, 84–91, 93–103, 105–107, 109–116, 118–123, 125). For data collection within synchronous and asynchronous HPV testing studies, some studies did not specify an HPV infection sample collection method, so the validity and reliability were unknown or they relied on self-reported HPV infections (n=10/92, 11%) (4, 33, 40, 55, 63, 64, 73, 94, 99, 101). For the last criteria, withdrawals and dropouts, few cohort studies described the number of and/or reasons for participants being lost-to-follow-up (n=8/33, 24%) (73, 74, 78–81, 83, 108).

## Discussion

After an expansive search of four databases for studies of dual-site oral and cervical HPV infections/cancers, we included 114 papers that evaluated the sites synchronously (n=80) (4, 15–93), asynchronously (n=12) (94–105), or by cancer diagnoses only (n=22) (106–127). This systematic review enhances the previous meta-analysis (4) by including more publication years, comprehensive search terms, databases, general oral HPV testing approaches, and formal study quality assessments of included studies using QATQS. We found that studies evaluating both oral and cervical HPV infections had cervical HPV+ rates that were higher than oral HPV+ rates.

The reporting of dual-site oral and cervical HPV infection rates was wide-ranging. On average, 15% of infections occurred concurrently in the oral cavity/oropharynx and cervix. Among concurrent oral-cervical HPV+ cases, HPV types were concordant across the two sites in an average of 41% of women. Asynchronous dual-site (oral-cervical) HPV infection rates also varied, spanning from 1.1% to 100%, with an average of 26% of study populations testing positive for both oral and cervical HPV at different times. Oral-cervical HPV type concordance was either very low (2%) or high (100%) for these asynchronously tested and dually-infected women, producing an average concordance rate of 39%. Combining synchronous and asynchronous oral-cervical HPV testing data, it was estimated that 16% of women were dually infected and 41% of the dually infected women had at least one concordant HPV type across sites. Most cancer diagnoses only studies reported an increased risk for a secondary cervical and/or oral cancer, resulting in incidence spanning 1.0-192.5/10,000 women. Regardless of timing, most studies were cross-sectional (n=79, 69%) (4, 15–72, 84–103) and therefore achieved an overall moderate rating with QATQS scoring criteria (n=81, 71%) (4, 15–26, 28, 29, 31–49, 52–58, 60, 62–72, 74, 75, 78, 82–91, 93–103, 112, 117, 119, 120).

Oral HPV infection can be especially difficult to detect which may explain the lower oral-cervical HPV type concordance rates or lack of significant findings in the reviewed studies. Saliva continuously rinses the mouth so it may aid in regional virus clearance, making oral HPV more transient than HPV infections at other sites. Most people clear oral HPV infections, often in as little as a few months, which means it can easily be missed (3). HPV detection in the oral cavity is not indicative of oropharyngeal cancer either. The virus tends to inhabit the oropharynx (e.g., tonsils), so if only buccal samples are being tested, HPV may go undetected. Rinsing or gargling within the oral cavity may only partially capture any HPV inhabiting the oropharyngeal region (3, 9, 99, 128). HPV testing materials were originally designed for cervical HPV; although repeatedly shown to be capable of HPV detection at other sites, materials might not be as effective at identifying oral HPV (94, 129). Many existing oral cancer diagnostic tests are questionable, lacking standard diagnostic protocols. New diagnostic approaches are evolving but are not yet validated (3). With the increase in oral HPV cancers, oral HPV sampling and testing methodology should improve over time.

Other reasons for non-significant findings within studies might be site-independent or biological in nature. Virus detection methods (e.g., assay, technique) vary in sensitivity levels and are often HPV type specific, so the chosen HPV test may not be able to detect the HPV type present, suggesting no infection (98–100, 103, 129–131). Poor or inappropriate sample collections at either site might also hinder a positive HPV result (98). HPV-infected, but otherwise healthy people can test negative for the virus and/or may develop HPV type-specific immunity at other uninfected mucosal sites (96, 98, 132). It is also biologically plausible for a cervical HPV+ woman to not be oral HPV+ given that cervical-oral HPV transmission between heterogeneous partners is common, but oral-oral HPV transmission is infrequent (94).

The current systematic review expands upon the narrowly-focused topic-related meta-analysis of 2010 (4) with the inclusion of additional oral-cervical HPV studies and their quality assessments. We identified a significant gap in the oral-cervical HPV literature with HPV type concordance between sites being understudied, highlighting the need for better HPV data collection and reporting efforts. HPV type was frequently missing. Synchronous studies usually provided HPV type data for one site and only HPV+/- status for the other site, despite data for both sites being collected concurrently. Asynchronous studies recruiting women with (pre)existing HPV conditions tended to only report basic HPV status for the secondary anatomical site. Cancer diagnoses only or registry-based studies did not collect any HPV type information. When HPV types were reported, data tended to be presented at aggregate levels with either totals or broad categories by anatomical site and/or HPV type (e.g., HPV+/- status only, HPV16/not HPV16, oncogenic/not oncogenic, groups of multiple HPV types).

Additional problems with vague data reporting were observed, irrespective of whether or not studies involved synchronous or asynchronous oral and cervical HPV testing or cancer diagnoses only. Many studies provided a general summary statement regarding the oral-cervical HPV relationship across sites with the corresponding statistics (e.g., odds ratios, (Cohen) kappa statistics, p-values). Enumeration of sub-sites of oral cancers made it difficult to calculate incidence consistently among cancer diagnoses only papers. Information on HPV type was represented as ranges or in bar graphs, which made it difficult to extract specific values and interpret results. Still others did not stratify oral HPV results by sex so cervical HPV data could not be cross-compared with respective oral samples in females.

Without specific HPV type information presented at the person-level for both the oral cavity/oropharynx and cervix, concurrent/dual-site versus concordant infections could not be elucidated. Additionally, not all dual-site HPV+ studies, especially asynchronous and cancer diagnoses only papers, discussed the potential for concurrent infections which made it difficult to determine if the identified oral and cervical HPV infections could be related. Few studies listed participant data individually, making it unclear if a participant had the same infection in both sites. In turn, oral-cervical HPV type concordance data was missing or could not be deduced for more than half of the papers (n=62/114, 54%) (17–19, 22, 24, 28, 30, 34, 35, 40, 41, 44–46, 48, 49, 53–55, 64, 66, 71, 75, 76, 79–83, 87, 90, 94, 95, 98–104, 106–127).

Lack of HPV type details also made it difficult to describe the oral-cervical HPV infection epidemiology more generally. About 10% of synchronous and asynchronous studies quantified the number of HPV infections (*vs*. HPV+ women) to account for multiple infections in women, which is an important detail to note, but complicated the estimation of the disease burden. More than 10% of synchronous and asynchronous studies did not discuss if any detected HPV types were high-risk. The interpretation of cancer diagnoses only papers could not be compared collectively with synchronous and asynchronous papers due to different effect estimates being used (i.e., SIR). Additionally, almost one-third of cancer diagnoses only papers (n=7/22, 32%) were missing SIR values (118–120, 122–125).

The current systematic review also had its limitations. Unpublished works and conference abstracts were excluded, potentially missing some information, however, we evaluated many peer-reviewed publications with broad search terms. Only papers written and published in English were included so there could be a lack of generalizability to international research. However, 94 international studies were captured with our search criteria (or 82.5% of all papers included in this systematic review were conducted outside the US). The inability to decipher the vagueness in oral cancer types (i.e., HPV *vs*. tobacco/alcohol related) and/or a lack of differentiation between HPV infection sites (e.g., oral-cervical data combined within multi-site results) potentially prevented some topic-relevant papers from being included in the current review. Regardless, studies had to justify oral cancers as potentially HPV-related to be included. Using strict review criteria, the current review included studies focused on HPV-related oral and cervical infections/cancers only, minimizing misclassification bias concerns. The systematic review also relied on literature-reported “oral HPV-related cancer” terminology to portray results. Inconsistent use of varying terms across publications impeded the summarization of results across studies. The standardized QATQS tool could not be fully utilized due to the topic-related nature of this systematic review relying only on observational studies.

To better understand the epidemiology of oral HPV transmission moving forward, data collection efforts need to be improved to include standardized reporting of HPV type data. Individual-level, site-specific HPV type data should be reported for every sample evaluated, especially when investigators are already using HPV DNA tests that provide such detailed information. Cancer diagnoses studies/registries should include a repository of HPV-evaluated bio-specimens such that site-specific HPV types can be identified. Broad categories, aggregated data, summary statistics, and analyses without stratum-specific results only provide an overview of a potential association of HPV infections/cancers across sites without being able to hone in on possible transmission routes which can only be divulged if HPV types are compared.

Detailed documentation of the timing of HPV site-specific sampling and evaluations are also needed to aid in determining concurrent HPV infections or the likelihood of an association between dual/multi-site HPV infections. Generation of a special access database to pool this person-level, site-specific HPV infection/cancer data would facilitate the additional analyses needed to understand the epidemiology of HPV transmission between sites. Better understanding site-specific HPV infection/cancer biology, including transmission routes, can further aid in preventing and minimizing future disease burdens.

In conclusion, few reviewed studies utilized strong epidemiological methodologies to determine HPV type concordance in dual-site oral and cervical infections. The results from this systematic review are inconclusive given the heterogeneity of included studies with wide-ranging oral-cervical HPV infection/cancer rates. Cervical HPV+ infection/cancer diagnoses tended to be more prevalent in women than oral HPV+ infections/cancers were. Given that these dual-site infection rates can vary significantly by female population and no oral HPV+ cancer screening approach exists, oral HPV+ cancer incidence may continue to increase unchecked. Additional studies identifying specific HPV infection types, both concurrently and over time, at multiple biological sites (especially oral and cervical, but also vaginal, vulval, penial, and anal) within women and men are needed to better understand how HPV is transmitted and determine any relationships between potentially HPV-related cancer sites. Pooling of these individual-level study results into a special access HPV database could facilitate future research investigations. From there, risk factors and populations with potentially increased oral and/or cervical HPV cancer risks could more easily be identified and incorporated into future public health prevention and control efforts, locally and globally, to reduce the HPV-related cancer burden in men and women.

## Data Availability Statement

The original contributions presented in the study are included in the article/**Supplementary Material**. Further inquiries can be directed to the corresponding author.

## Author Contributions

All authors agree to be accountable for the content of the work. KJ: validation, formal analysis, investigation, data curation, writing-original draft, writing-review and editing, and project administration. CB: validation, formal analysis, investigation, data curation, writing-original draft, writing-review and editing, and visualization. XZ: validation, formal analysis, investigation, data curation, writing-original draft, and writing-review and editing. EP: conceptualization, methodology, resources, writing-review and editing, and supervision. All authors contributed to the article and approved the submitted version.

## Funding

KJ (P01 CA229143-S1) and XZ (F99CA253745-01) both have National Cancer Institute (NCI) funding through the National Institutes of Health (NIH). CB is funded through The Ohio State University Comprehensive Cancer Center.

## Conflict of Interest

EP receives grant funding through the university from Pfizer and Merck Foundation.

The remaining authors declare that the research was conducted in the absence of any commercial or financial relationships that could be construed as a potential conflict of interest.

## Publisher’s Note

All claims expressed in this article are solely those of the authors and do not necessarily represent those of their affiliated organizations, or those of the publisher, the editors and the reviewers. Any product that may be evaluated in this article, or claim that may be made by its manufacturer, is not guaranteed or endorsed by the publisher.
