# Supplementary material for: Low Rates of Dual-Site and Concordant Oral-Cervical Human Papillomavirus Infections and Cancers: A Systematic Review
Source: Front Oncol. 2022 Mar 29;12:848628. doi: 10.3389/fonc.2022.848628 (PMC9004260; doi:10.3389/fonc.2022.848628)
Supplement: Supplementary file 1 [file DataSheet_1.docx]

Supplementary Table. Additional Database-specific Search Terms for “Oral-cervical HPV Infections/Cancers: A Systematic Review”.

| Database | Search Terms | Filters |
| --- | --- | --- |
| EMBASE | (cervix OR cervical OR cerv*) AND (head AND neck OR oral OR oropharyngeal OR oropharynx OR oropharyn* OR (soft AND palate) OR esophagus OR esophageal OR esophag* OR hypopharynx OR hypopharyngeal OR hypopharyn* OR larynx OR laryngeal OR laryn* OR nasopharyngeal OR nasopharynx OR nasopharyn* OR tonsil OR tonsillar OR tonsil* OR throat) AND (hpv OR (human AND papilloma AND virus) OR papillomaviridae OR (human AND papillomavirus)) AND (cancer OR cancerous OR cancer* OR carcinoma OR carcinom* OR neoplasia OR neoplasm OR neoplas* OR tumor OR tumor* OR tumorous OR dysplasia OR (intra AND epithelial AND neoplasia) OR (intra AND epithelial AND dysplasia) OR (mucosal AND lesion) OR infection OR infect* OR malignancy OR malignant OR malignan* OR (precancerous AND lesion) OR (pre AND cancerous AND lesion) OR (squamous AND cell AND carcinoma)) AND [embase]/lim AND [1990-2019]/py AND [english]/lim AND [humans]/lim | (applied within search terms) |
| Ovid Medline | (Cervix or cervical or cerv*).af.  ((head and neck) or oral or oropharyngeal or oropharynx or oropharyn* or (soft and palate) or esophagus or esophageal or esophag* or hypopharynx or hypopharyngeal or hypopharyn* or larynx or laryngeal or laryn* or nasopharyngeal or nasopharynx or nasopharyn* or tonsil or tonsillar or tonsil* or throat).af.  (hpv or (human and papilloma and virus) or papillomaviridae or (human and papillomavirus)).af.  (cancer or cancerous or cancer* or carcinoma or carcinom* or neoplasia or neoplasm or neoplas* or tumor or tumor* or tumorous or dysplasia or (intra and epithelial and neoplasia) or (intra and epithelial and dysplasia) or (mucosal and lesion) or infection or infect* or malignancy or malignant or malignan* or (precancerous and lesion) or (pre and cancerous and lesion) or (squamous and cell and carcinoma)).af. | English,  Humans,  yr=”1990-2019” |
| Web of Science | #1: (ALL= (Cervix) OR ALL= (cervical) OR ALL= (cerv*))  #2: ALL = (head and neck) OR ALL = (oral) OR ALL = (oropharyngeal) OR ALL = (oropharynx) or ALL = (oropharyn*) OR ALL = (soft and palate) OR ALL = (esophagus) or ALL = (esophageal) or ALL = (esophag*) OR ALL = (hypopharynx) or ALL = (hypopharyngeal) or ALL = (hypopharyn*) OR ALL = (larynx) or ALL = (laryngeal) or ALL = (laryn*) OR ALL = (nasopharyngeal) OR ALL = (nasopharynx) or ALL = (nasopharyn*) Or ALL = (tonsil) or ALL = (tonsillar) or ALL = (tonsil*) OR ALL = (throat)  #3: ALL= (hpv) or ALL= (human and papilloma and virus) or ALL= (papillomaviridae) or ALL= (human and papillomavirus)  #4: ALL = (cancer) or ALL = (cancerous) or ALL = (cancer*) OR ALL = (carcinoma) or ALL = (carcinom*) OR ALL = (neoplasia) OR ALL = (neoplasm) OR ALL = (neoplas*) OR ALL = (tumor) or ALL = (tumor*) or ALL = (tumorous) OR ALL = (dysplasia) OR ALL = (intra and epithelial and neoplasia) or ALL = (intra and epithelial and dysplasia) OR ALL = (mucosal and lesion) OR ALL = (infection) or ALL = (infect*) OR ALL = (malignancy) OR ALL = (malignant) or ALL = (malignan*) OR ALL = (precancerous and lesion) OR ALL = (pre and cancerous and lesion) OR ALL = (squamous and cell and carcinoma)  #5: #1 AND #2 AND #3 AND #4  #6: TS = (animal* AND model*) OR TS = (animal* AND tissue*) OR TS = (animal* AND experiment*) OR TS = (animal* AND cell*) OR TS = (mouse) OR TS = (mice)  #7: #5 NOT #6  #8: (#7) *AND***LANGUAGE:** (English)  #9: #8, *Timespan=1990-2019* | (applied within search terms) |
